# ADMETlab: a platform for systematic ADMET evaluation based on a comprehensively collected ADMET database

**DOI:** 10.1186/s13321-018-0283-x

**Published:** 2018-06-26

**Authors:** Jie Dong, Ning-Ning Wang, Zhi-Jiang Yao, Lin Zhang, Yan Cheng, Defang Ouyang, Ai-Ping Lu, Dong-Sheng Cao

**Affiliations:** 1 Xiangya School of Pharmaceutical Sciences, Central South University, No. 172, Tongzipo Road, Yuelu District, Changsha, People’s Republic of China; 2 Hunan Key Laboratory of Grain-oil Deep Process and Quality Control, College of Food Science and Engineering, National Engineering Laboratory for Deep Processing of Rice and Byproducts, Central South University of Forestry and Technology, Changsha, People’s Republic of China; 3 Hunan Key Laboratory of Processed Food for Special Medical Purpose, Central South University of Forestry and Technology, Changsha, People’s Republic of China; 4 Institute for Advancing Translational Medicine in Bone & Joint Diseases, School of Chinese Medicine, Hong Kong Baptist University, Hong Kong SAR, People’s Republic of China; 5 State Key Laboratory of Quality Research in Chinese Medicine, Institute of Chinese Medical Sciences (ICMS), University of Macau, Macau, People’s Republic of China

**Keywords:** ADMETlab, ADMET, Drug-likeness, ADMET database, Drug discovery, Cheminformatics

## Abstract

Current pharmaceutical research and development (R&D) is a high-risk investment which is usually faced with some unexpected even disastrous failures in different stages of drug discovery. One main reason for R&D failures is the efficacy and safety deficiencies which are related largely to absorption, distribution, metabolism and excretion (ADME) properties and various toxicities (T). Therefore, rapid ADMET evaluation is urgently needed to minimize failures in the drug discovery process. Here, we developed a web-based platform called ADMETlab for systematic ADMET evaluation of chemicals based on a comprehensively collected ADMET database consisting of 288,967 entries. Four function modules in the platform enable users to conveniently perform six types of drug-likeness analysis (five rules and one prediction model), 31 ADMET endpoints prediction (basic property: 3, absorption: 6, distribution: 3, metabolism: 10, elimination: 2, toxicity: 7), systematic evaluation and database/similarity searching. We believe that this web platform will hopefully facilitate the drug discovery process by enabling early drug-likeness evaluation, rapid ADMET virtual screening or filtering and prioritization of chemical structures. The ADMETlab web platform is designed based on the Django framework in Python, and is freely accessible at http://admet.scbdd.com/.

**Electronic supplementary material:**

The online version of this article (10.1186/s13321-018-0283-x) contains supplementary material, which is available to authorized users.

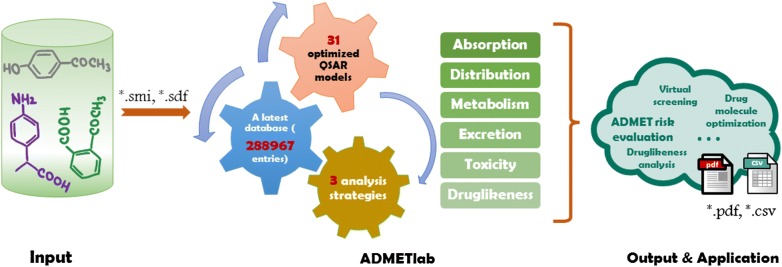

## Background

Current pharmaceutical research and development is a high-risk investment that is characterized by a complex process including disease selection, target identification, lead discovery and optimization, as well as preclinical and clinical trials. Although millions of active compounds have been found, the number of new drugs approved didn’t increase drastically in recent years [1–3]. Besides the non-technical issues, the efficacy and safety deficiencies could account for the main stagnation which is related largely to absorption, distribution, metabolism and excretion (ADME) properties and various toxicities (T). ADME covers the pharmacokinetic issues determining whether a drug molecule will get to the target protein in the body, and how long it will stay in the bloodstream. Parallel evaluation of efficiency and biopharmaceutical properties of drug candidates has been standardized, and exhaustive studies of ADMET processes are nowadays routinely carried out at early stage of drug discovery to reduce the attrition rate. This is because the majority of clinical trial failures have been due to ADMET issues, not from a lack of efficacy. Since this is the most costly point to have a failure, ADMET-related research could save much time and money if they can divert even one clinical trial failure [4, 5]. Moreover, the current experimental methods for ADMET evaluation are still costly and time-consuming, and they need a lot of animal testing which is usually inadequate when managing hundreds of compounds in the early stage of drug discovery. In order to minimize failures, computational strategies are sought by medicinal chemists to predict the fate of drugs in organism, and to early identify the risk of toxicity [6, 7]. ADMET-related in silico models are commonly used to provide a fast and preliminary screening of ADMET properties before compounds are further investigated in vitro [8–11]. Currently, there are several free and commercial computational tools for predicting ADMET properties. However, these tools are not yet very accurate. Moreover, most of existing computational tools are individual models which focus on specific ADMET properties and few can evaluate different ADMET properties simultaneously due to the limited data size and methods [12–14].

**Fig. 1:**
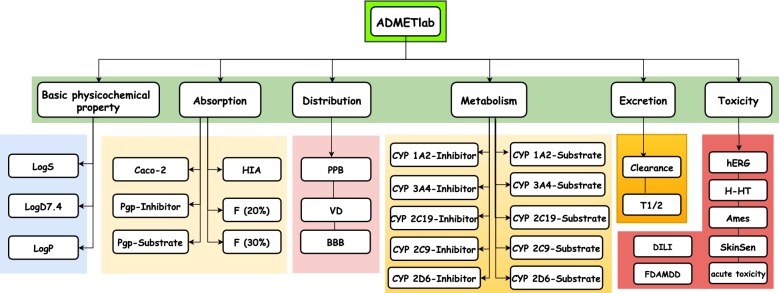
An overview of ADMET properties that can be evaluated by ADMETlab

In order to facilitate the ADMET evaluation, we developed a web platform called ADMETlab based on a comprehensively collected database which integrates the existing ADMET and basic physicochemical-related endpoints as many as possible (see Fig. 1). Four main modules are designed to conveniently assess ADMET properties: drug-likeness evaluation, ADMET prediction (31 endpoints assessment), systematic ADMET evaluation for single chemical and database/similarity searching based on ADMET database with 288,967 entries. Compared with other online platforms, our proposed ADMETlab incorporated more ADMET endpoints and improved model performance for some endpoints based on large and structurally diverse data sets. These modules are deployed in a user-friendly, freely available web interface (http://admet.scbdd.com/) and we recommend it as a valuable tool for medicinal chemists in the drug discovery process.

## Implementation

### Development environment

ADMETlab consists of two main components: “ADMET database” and “Web platform”. They share a common running environment. We deployed an elastic compute service (ECS) server of Aliyun to run the whole project. The number of CPU cores and memory are automatically allocated to the running instances on demand, which ensures the elastically stretchable computing capability. In this project, Python was chosen as the main programming language because of its considerable libraries for the scientific computation. We use *Python*-*RDKit* [15], *Pybel* to wrap molecules; [16] use *Chemopy* [17] *ChemDes* [18] *and BioTriangle* [19] to calculate molecular descriptors and fingerprints; use *Scikit*-*learn* to build models of different algorithms; [20] use *Numpy* [21], *Pandas* to wrap calculating results into numeric values or files [22]. Django is chosen as a high-level Python web framework which allows for the rapid development and clear design. According to its model visualization-control (MVC) design pattern, the whole system is divided into three main components: the backend calculating program, the back-end control program and the front-end visualization program. At the backend, uWSGI + Nginx worked as the web server software, The MySQL database was used for data storage and retrieval. It should be noted that ‘ADMET database’ and ‘Web platform’ shared a common database instance. At the front end, the website is designed in accordance with W3C standards based on HTML, CSS, and JavaScript languages.

### User interface

ADMETlab provides a convenient and easy-to-use interface for users. The user interface of ADMETlab consists of four main modules: “Webserver”, “Search”, “Documentation” and “Help”. “Webserver” is the main entrance for users to use “Web platform”, which includes three sub modules: “Druglikeness Evaluation”, “ADMET Prediction” and “Systematic Evaluation”. “Druglikeness Evaluation” module enables users to calculate 5 commonly used druglikeness rules and provides a druglikeness model. This model can not only find out the active compounds from chemical entities but also distinguish the potential drug candidates from active compounds. “ADMET Prediction” module provides 31 models to predict 31 ADMET related properties. Users need to choose one model to obtain results for one or multiple molecules, which is suitable for screening target molecules of a specific endpoint. “Systematic Evaluation” predicts all-sided pharmacokinetic properties of a specific promising compound and users will have an overall understanding of this compound. “Search” module is the interface for ADMET database, which enables users to perform accurate search, range search and similarity search. “Documentation” module provides detailed information about data, methodologies and results of ADMETlab. The “Help” module describes examples about how to use the ADMETlab platform.

### Input/output

The Input/Output system is mainly responsible for the input or output of the strings, commands and files. ADMETlab uses the functions like *file, open, write, getcwd* and *setcwd* from Python I/O system to accomplish the file reads and writes. For “Druglikeness Evaluation” and “ADMET Prediction” module, *SMILES* and *SDF* are acceptable molecular file types. These two modules provide three kinds of input ways: by inputting *SMILES*, by uploading files and by drawing molecules from the JME editor. The outputs of them are interactive data table and *CSV* file. The interactive data table for five rules contains evaluation values for each point; each of the items can be expanded to see the detailed information and structures. Interactive data table for the model prediction results contains predicted values and structures. All the data tables allow for searching and ranking by the values. For “Systematic Evaluation” module, *SMILES* is acceptable molecular format, and the output is rendered as *HTML* page which contains basic information about the query molecule and predicted values of all the endpoints. For “Search” module, the *SMILES* and related parameters are set for input; the output is rendered as *HTML* page which contains interactive data table of all satisfied items.

## Methods

### Data collection

The data of ADMETlab consisted of two parts. The first part was collected from peer-reviewed publications through manually filtering and processing. Note that this part will also be then used to the modeling process. The second part was collected from ChEMBL [23], EPA [24] and DrugBank databases [25]. The corresponding basic information and experimental values were collected at the same time. All the obtained data were checked and washed by molecular operating environment (MOE, version 2016) and then divided into six classes (basic, A, D, M, E and T) and a series of subclasses according to their endpoint meanings. After the format standardization and combination, 288,967 entries were obtained and then were input into the database. More detailed description can be found in the “Documentation” section of the website.

### Data set preparing

In the data collection process, we finally obtained 31 datasets for ADMET modeling from the first part of data. For these datasets, the following pretreatments were carried out to guarantee the quality and reliability of the data: (1) removing compounds that without explicit description for ADME/T properties; (2) for the classification data, reserve only one entity if there are two or more same compounds; (3) for the regression data, if there are two or more entries for a molecule, the arithmetic mean value of these values was adopted to reduce the random error when their fluctuations was in a reasonable limit, otherwise, this compound would be deleted; (4) Washing molecules by MOE (disconnecting groups/metals in simple salts, keeping the largest molecular fragment and add explicit hydrogen). After that, a series of high-quality datasets were obtained. According to the Organization for Economic Co-operation and Development (OECD) principles, not only the internal validation is needed to verify the reliability and predictive ability of models, but also the external validation [11]. Therefore, all the datasets were divided into training set and test set according to the chemical space distribution by “Diverse training set split” module from ChemSAR [26]. In this step, we set a threshold that 75% compounds were used as training set and the remaining 25% as test set. The detailed information for these datasets can be seen in Table 1.

**Table 1:** The statistical results of the datasets for modeling

Category	Property	Total	Positive	Negative	Train	Test
Basic physicochemical property	LogS	5220	–	–	4116	1104
	LogD_7.4_	1031	–	–	773	258
	LogP					
Absorption	Caco-2	1182	–	–	886	296
	Pgp-inhibitor	2297	1372	925	1723	574
	Pgp-substrate	1252	643	609	939	313
	HIA	970	818	152	728	242
	F (20%)	1013	759	254	760	253
	F (30%)	1013	672	341	760	253
Distribution	PPB	1822	–	–	1368	454
	VD	544	–	–	408	136
	BBB	2237	540	1697	1678	559
Metabolism	CYP 1A2-inhibitor	12,145	5713	6432	9145	3000
	CYP 1A2-substrate	396	198	198	297	99
	CYP 3A4-inhibitor	11,893	5047	6846	8893	3000
	CYP 3A4-substrate	1020	510	510	765	255
	CYP 2C9-inhibitor	11,720	3960	7760	8720	3000
	CYP 2C9-substrate	784	278	506	626	156
	CYP 2C19-inhibitor	12,272	5670	6602	9272	3000
	CYP 2C19-substrate	312	156	156	234	78
	CYP 2D6-inhibitor	12,726	2342	10,384	9726	3000
	CYP 2D6-substrate	816	352	464	611	205
Excretion	Clearance	544	–	–	408	136
	T_1/2_	544	–	–	408	136
Toxicity	hERG	655	451	204	392	263
	H-HT	2171	1435	736	1628	543
	Ames	7619	4252	3367	5714	1905
	Skin sensitivity	404	274	130	323	81
	Rat oral acute toxicity	7397	–	–	5917	1480
	DILI	475	236	239	380	95
	FDAMDD	803	442	361	643	160

### Descriptor calculation

In this part, molecular descriptors and fingerprints were applied to further model building. The molecular descriptors include 11 types of widely used descriptors: constitution, topology, connectivity, E-state, Kappa, basak, burden, autocorrelation, charge, property, MOE-type descriptors and 403 descriptors in total. All the descriptors were calculated by using Chemopy—a python package built by our group. These continuous descriptors were used to build regression models. The fingerprints include FP2, MACCS, ECFP2, ECFP4 and ECFP6, which were calculated by using *ChemDes* [18] and *BioTriangle* [19]. These fingerprints were used to build classification models. All descriptors were firstly checked to ensure that the values of each descriptor are available for molecular structures. The detailed information of these mentioned descriptors can be seen in Table 2.

**Table 2:** The molecular descriptors that were used in modeling process

Descriptor type	Description	Number
Constitution	Constitutional descriptors	30
Topology	Topological descriptors	35
Connectivity	Connectivity indices	44
E-state	E-state descriptors	79
Kappa	Kappa shape descriptors	7
Basak	Basak information indices	21
Burden	Burden descriptors	64
Autocorrelation	Morgan autocorrelation	32
Charge	Charge descriptors	25
Property	Molecular property	6
FP2	A path-based fingerprint which indexes small molecule fragments based on linear segments of up to 7 atoms	2048
MACCS	MACCS keys	167
ECFP2	An ECFP feature represents a circular substructure around a center atom with diameter is 1	2048
ECFP4	An ECFP feature represents a circular substructure around a center atom with diameter is 2	2048
ECFP6	An ECFP feature represents a circular substructure around a center atom with diameter is 3	2048

### Descriptor selection

To build those regression models, we need to select proper descriptors. Before further descriptor selection, three feature pre-selection steps were performed to eliminate some uninformative descriptors: (1) remove descriptors whose variance is zero or close to zero; (2) remove descriptors, the percentage of whose identical values is larger than 95% and (3) if the correlation of two descriptors is large than 0.95, one of them was randomly removed. The remaining descriptors were used to further perform descriptor selection and QSAR modeling. For these molecular descriptors, further descriptor selection need be carried out to eliminate uninformative and interferential descriptors. In this study, we utilize the internal descriptor importance ranking function in random forest (RF) to select informative descriptors [27]. The descriptor selection procedure is performed as follows: Firstly, all descriptors were applied to build a model. The number of *estimators* of RF was set as 1000; the *mtry* was set as $\sqrt {\text{p}}$, the other parameters were set as defaults, and fivefold cross-validation was used to evaluate the model. These involved descriptors were sorted according to their importance, and then the last two descriptors were removed and the rest were used to rebuild the model and a new descriptor order was obtained. Repeat this step until the last two remaining descriptors were left, and at last we get a series of models based on different numbers of descriptors. Among them, we can choose a best feature combination according to the number of descriptors and the error value of the model.

### Modeling algorithms

In this study, six different modeling algorithms were applied to develop QSAR regression or classification models for ADME/T related properties: random forests (RF), support vector machine (SVM), recursive partitioning regression (RP), partial least square (PLS), naïve Bayes (NB) and decision tree (DT).

RF is an ensemble of unpruned classification or regression trees created by using bootstrap samples of the training data and random feature selection in tree induction, which was firstly proposed by Breiman in 2001 [28, 29]. SVM is an algorithm based on the structural risk minimization principle from statistical learning theory. Although developed for classification problems, SVM can also be applied to the case of regression [30]. RP has been developed since the 1980s and it is a statistical method for multivariable analysis. RP creates a decision tree that strives to correctly classify members of the population by splitting it into sub-populations based on several dichotomous independent variables. The process is termed recursive because each sub-population may in turn be split an indefinite number of times until the splitting process terminates after a particular stopping criterion is reached [31]. PLS is a recently developed generalization of multiple linear regression (MLR), it is of particular interest because, unlike MLR, it can analyze data with strongly collinear, noisy, and numerous X-variables, and also simultaneously model several response variables [32, 33]. NB is a simple learning algorithm that utilizes Bayes rule together with a strong assumption that the attributes are conditionally independent, given the class. Coupled with its computational efficiency and many other desirable features, NB has been widely applied in practice [34]. DT is a non-parametric supervised learning method used for classification and regression. The goal is to create a model that predicts the value of a target variable by learning simple decision rules inferred from the data features [35]. Among these six methods, the RF, SVM, RP and PLS were used for regression model building; the RF, SVM, NB and DT were applied to build those classification models. Before the modeling building, all related parameters of some algorithms should be optimized. They are (*estimators, mtry*) for RF, (*Sigma, C*) for SVM (*rbf*) and (*n_components*) for PLS separately. The cross validation method based on grid search was adopted to obtain optimized parameter sets. Specifically, for RF we tried the *estimators* of 500 and 1000; the *mtry* was optimized through two stages: firstly, use 20 as the *step length* and (1, *n_features*) as the *range*, and then use 2 as the *step length* and (*mtry*ʹ − 50, *mtry*ʹ + 50) as the range while *mtry*ʹ − 50 > 0 and *mtry*ʹ + 50 ≤ *n_features.* The *mtry*ʹ was the result of stage 1. Similarly, for SVM (*rbf*) two stages were applied to optimize the parameter sets. Firstly, the coarse grid-search process used: *C* = {*start*: 2^(− 5), *end*: 2^(15), *step*: 2^(2)} and *Sigma* = {*start*: 2^(− 15), *end*: 2^(3), *step*: 2^(2)}. Secondly, the finer grid-search process used 2^(0.25) as the *step length* to optimize the results from stage 1. For PLS, the best *n_components* was optimized from 1 to 100.

For some unbalanced datasets, the obtained models may be biased if general modeling processes were applied. To obtain some more balanced classification models, we proposed two new methods to achieve this goal: (1) Samplesize parameter in RF. When this parameter is set to 100, it means that 100 positive compounds and 100 negative compounds were randomly selected to build a tree in each modeling process and this process repeated many times to guarantee that every compound in the training set could be used in the final RF model. The use of this method guarantees that the number of positive samples and negative samples is relatively balanced in each bootstrap sampling process. (2) The random sampling method was applied for the positive compounds (if positive samples are much more than negative samples) in each modeling process and this process was repeated 10 times. Finally, a consensus model was obtained for further application based on these 10 classification models. Besides, The Cohen’s kappa coefficient can be used as a performance metric to evaluate the results of models based on unbalanced dataset. Here we calculated the coefficient for the 7 unbalanced models (see the “Documentation”). Considering the barely satisfactory results of some properties such as VD, CL, T_1/2_ and LD_50_ of acute toxicity, the percentage of compounds predicted within different fold errors (Folds) was applied to assess model performance. They are defined as follows: fold = 1 + |Y_pred_ − Y_true_|/Y_true_. A prediction method with an average-fold error < 2 was considered successful.

### Performance evaluation

To ensure the obtained QSAR model has good generalization ability for a new chemical entity, fivefold cross-validation and a test set were applied for this purpose. For fivefold cross-validation, the whole training set was split into five roughly equal-sized parts firstly. Then the model was built with four parts of the data and the prediction error of the other one part was calculated. The process was repeated five times so that every part could be used as a validation set. For these regression models, six commonly used parameters were applied to evaluate their quality: the square correlation coefficients of fitting (R_F_^2^); the root mean squared error of fitting (RMSE_F_); the square correlation coefficients of cross-validation (Q^2^); the root mean squared error of cross validation (RMSE_cv_), the square correlation coefficients of test set (R_T_^2^); the root mean squared error of test set (RMSE_T_). As to these classification models, four parameters were proposed for their evaluation: accuracy (ACC); specificity (SP); sensitivity (SE); the area under the ROC curve (AUC). Their statistic definitions are as follows:

$$R_{F}^{2} = 1 - \frac{{\sum \left( {\hat{y}_{i} - y_{i} } \right)^{2} }}{{\sum (y_{i} - \bar{y})^{2} }}$$

$$RMSE_{F} = \sqrt {\frac{1}{N}\mathop \sum \limits_{1 = 1}^{N} \left( {y_{i} - \hat{y}_{i} } \right)^{2} }$$

$$Q^{2} = 1 - \frac{{\sum \left( {\hat{y}_{\left( v \right)i} - y_{i} } \right)^{2} }}{{\sum (y_{i} - \bar{y})^{2} }}$$

$$RMSE_{cv} = \sqrt {\frac{1}{N}\mathop \sum \limits_{1 = 1}^{N} \left( {y_{i} - \hat{y}_{\left( v \right)i} } \right)^{2} }$$

$$R_{T}^{2} = 1 - \frac{{\sum \left( {\hat{y}_{i} - y_{i} } \right)^{2} }}{{\sum (y_{i} - \bar{y})^{2} }}$$

$$RMSE_{T} = \sqrt {\frac{1}{N}\mathop \sum \limits_{1 = 1}^{N} \left( {y_{i} - \hat{y}_{i} } \right)^{2} }$$

$$ACC = \frac{{{\text{TP}} + {\text{TN}}}}{{{\text{TP}} + {\text{TN}} + {\text{FP}} + {\text{FN}}}}$$

$$SP = \frac{\text{TN}}{{{\text{TN}} + {\text{FP}}}}$$

$$SE = \frac{\text{TP}}{{{\text{TP}} + {\text{FN}}}}$$

where $\hat{y}_{i}$ and $y_{i}$ are the predicted and experimental values of the *i*th sample in the data set; $\bar{y}$ is the mean value of all the experimental values in the training set; $\hat{y}_{\left( v \right)i}$ is the predicted value of *i*th sample for cross validation; *N* is the number of samples in the training set. TP, FP, TN and FN represent true positive, false positive, true negative and false negative, respectively.

## Results and discussion

### Drug-likeness analysis

This drug-likeness analysis module is designed for users to filter those chemical compounds that are not likely to be leads or drugs. The module includes five commonly used drug-likeness rules (Lipinski, Ghose, Oprea, Veber, and Varma) and one well-performed classification model [36–40]. The classification model consisting of 6731 positive samples from DrugBank and 6769 negative samples from ChEMBL with IC50 or Ki values < 10 μm was constructed based on the random forest method and MACCS fingerprint, with classification accuracy of 0.800 and AUC score of 0.867 by external test set. By means of drug-likeness analysis, users can preliminarily screen out some promising compounds that are likely to be leads or drugs in the early stage of drug discovery.

### ADMET prediction

To quickly evaluate various ADMET properties, a series of high-quality prediction models were generated and validated. Totally, there are 9 regression models (LogP was from RDKit directly) and 22 classification models with improved performance in this platform (basic property: 3, absorption: 6, distribution: 3, metabolism: 10, elimination: 2, toxicity: 7). Different methods, different representations and large datasets, to our best knowledge, were applied to obtain these optimal models (see Additional file 1). For some unbalanced datasets (e.g., HIA, CYP2C9-Substrate, CYP2D6-Substrate) or hard-to-predict endpoints (e.g., CL, T1/2, acute toxicity), several useful strategies were proposed to improve prediction ability of models (see Additional file 1). For example, resampling strategy and ensemble techniques are applied to cope with those unbalanced data. The parameter adjusting class balance in the random forest algorithm is optimized to obtain balanced models. For each property, the detailed explanation and corresponding suggestion are provided for users to give a meaningful understanding of prediction results. This module allows the batch prediction and users can realize rapid ADMET screening or filtering based on these specific prediction models.

The performances of the models are shown in Tables 3, 4 and 5. From the results we can see: (1) Most of the models obtained a good performance; LogS, LogD_7.4_ and Caco-2 got a Q^2^ > 0.84; 86% of the classification models got accuracy > 0.7; 50% of the classification models got accuracy > 0.8. All the models had a better or comparable performance compared with previous works in peer-reviewed publications, which was discussed in detail in the Additional file 1. (2) There were still few models got a low Q^2^ or accuracy like PPB, VD, F20 and F30, while these models have been also improved by using larger dataset or good modeling strategies compared with previous published ones. (3) For obvious unbalanced datasets: F20, F30, CYP2C9-Substrate and CYP2D6-Substrate, their best performance models were not the same with those in Table 5. From the results in Additional file 1 we found that the SE was about twice as much as SP, which led to an ineffective classifier. This phenomenon was caused by the unbalanced datasets. After it was processed with the strategies mentioned above, the SE and SP became very close. To F20, the SE/SP of the best model was optimized to 0.731/0.647 (RF + MACCS) from 0.907/0.450 (SVM + MACCS). The F30, CYP2C9-Substrate and CYP2D6-Substrate were also improved by this way. From the results of Cohen’s kappa coefficient, we can see that after the processing using our strategies, the consistency is quite acceptable. 4) RF method showed a best ability to build regression models of datasets in Tables 3 and 4; SVM and RF methods combined with ECFP4 performed best in most cases in datasets of Table 5.

**Table 3:** The best regression models for some ADMET related properties (Part 1)

Property	Method	mtry	R^2^	Q^2^	R_T_^2^	RMSE_F_	RMSE_CV_	RMSE_T_
LogS	RF	10	0.980	0.860	0.979	0.095	0.698	0.712
LogD_7.4_	RF	14	0.983	0.877	0.874	0.228	0.614	0.605
Caco-2	RF	14	0.973	0.845	0.824	0.121	0.289	0.290
PPB	RF	8	0.954	0.691	0.682	7.124	18.443	18.044
VD	RF	10	0.950	0.634	0.556	0.281	0.762	0.948

**Table 4:** The best regression models for some ADMET related properties (Part 2)

Property	Method	Features	mtry	Twofold rate (CV/test)	Threefold rate (CV/test)
CL	RF	2D	10	0.760/0.816	0.877/0.897
T_1/2_	RF	2D	12	0.762/0.699	0.897/0.824
LD50	RF	2D	5	0.986/0.987	0.998/0.997

**Table 5:** The best classification models for some ADME/T related properties

Property	Method	Features	Fivefold cross validation				External validation dataset			
			Sensitivity	Specificity	Accuracy	AUC	Sensitivity	Specificity	Accuracy	AUC
HIA	RF	MACCS	0.820	0.743	0.782	0.846	0.801	0.743	0.773	0.831
F (20%)	RF	MACCS	0.731	0.647	0.689	0.759	0.680	0.663	0.671	0.746
F (30%)	RF	ECFP6	0.743	0.605	0.669	0.715	0.751	0.601	0.667	0.718
BBB	SVM	ECFP2	0.962	0.813	0.926	0.948	0.993	0.854	0.962	0.975
Pgp-inhibitor	SVM	ECFP4	0.887	0.789	0.848	0.908	0.863	0.802	0.838	0.913
Pgp-substrate	SVM	ECFP4	0.839	0.807	0.824	0.899	0.826	0.854	0.840	0.905
CYP1A2-inhibitor	SVM	ECFP4	0.833	0.864	0.849	0.928	0.853	0.880	0.867	0.939
CYP1A2-substrate	RF	ECFP4	0.768	0.636	0.702	0.801	0.768	0.637	0.702	0.802
CYP3A4-inhibitor	SVM	ECFP4	0.759	0.858	0.817	0.901	0.788	0.860	0.829	0.909
CYP3A4-substrate	RF	ECFP4	0.798	0.716	0.757	0.835	0.819	0.679	0.749	0.835
CYP2C19-inhibitor	SVM	ECFP2	0.826	0.819	0.822	0.893	0.812	0.825	0.819	0.899
CYP2C19-substrate	RF	ECFP2	0.735	0.744	0.740	0.816	0.871	0.667	0.769	0.853
CYP2C9-inhibitor	SVM	ECFP4	0.719	0.898	0.837	0.900	0.730	0.882	0.830	0.894
CYP2C9-substrate	RF	ECFP4	0.746	0.709	0.728	0.819	0.746	0.709	0.734	0.824
CYP2D6-inhibitor	RF	ECFP4	0.770	0.811	0.793	0.868	0.771	0.812	0.795	0.882
CYP2D6-substrate	RF	ECFP4	0.765	0.73	0.748	0.823	0.792	0.73	0.76	0.833
hERG	RF	2D	0.908	0.700	0.844	0.879	0.888	0.762	0.848	0.873
H-HT	RF	2D	0.780	0.520	0.689	0.710	0.785	0.487	0.681	0.683
Ames	RF	MACCS	0.800	0.841	0.820	0.890	0.848	0.816	0.834	0.897
SkinSen	RF	MACCS	0.685	0.727	0.706	0.760	0.715	0.727	0.731	0.774
DILI	RF	MACCS	0.866	0.813	0.840	0.904	0.830	0.857	0.843	0.910
FDAMDD	RF	ECFP4	0.848	0.812	0.832	0.904	0.853	0.782	0.821	0.892

### Systematic ADMET evaluation

For a specific compound, this module provides a convenient tool for systematic ADMET evaluation by predicting all-sided pharmacokinetic properties and thus users will have an overall understanding of ADMET properties of this compound. By inputting a molecule, “Predicted values”, “Probability”, “Suggestion”, “Meaning & Preference” and “Reference” will be shown according to different endpoints. For regression models the “Predicted values” is shown as numeric values with commonly used units. For classification models the number of “+” or “−” were used to represent the “Predicted values” according to the “Probability”. This will give a more clear and intuitive representation instead of a numeric character. For each endpoint, the reasonable recommendation (“Suggestion”) for ADMET is also provided. According to these given suggestion, users can extract some rational compounds with multiple reasonable profiles and further optimize their chemical structures in a purposeful way to make them more potential to be drugs. Besides, the “Meaning & Preference” summarizes the key points of knowledge-based rules for each endpoint and category standards from the “Reference”. This strongly assists researchers to evaluate ADMET of the specific compound in a systematic way.

### Database searching

Based on the comprehensive ADMET database, the database searching and similarity searching were provided for users. With an input of molecular structures or pharmacokinetic properties, the matched compounds in the database can be listed in the result table. For the basic searching, two approaches are provided: accurate searching by SMILES, CAS registry number or IUPAC name; range searching via the range of molecular weight, AlogP, hydrogen bond acceptor or hydrogen bond donor. For similarity searching, different structural similarity criterions can be chosen to search similar compounds to the input structure. Here, we provide five kinds of fingerprints to represent molecular information and two kinds of similarity metrics for similarity search. According to these results, users can not only evaluate ADMET properties for a new compound but also obtain some useful hints about its structure optimization.

### Features

Currently, there have been several tools that contribute to ADMET analysis in different ways. However, ADMETlab has some unique and good features: (1) Providing a largest database containing direct ADMET data values. The database collected 288,967 entries from different data sources, each of which not only records the “ADMET values”, “Class”, “Subclass” and “Structure” but also 18 annotations like “IUPACName”, “Description” and “Reference”. (2) Comparative large datasets of most properties. For modeling of each property, the datasets was manually collected and integrated from reliable peer-reviewed publications and databases as many as possible. This guarantees a large and structurally diverse dataset and the broader application domain than other ones. (3) Better and robust SAR/QSAR models. For each endpoint, we employed different algorithms combined with different representations and obtained comparable or better models than other tools which have been discussed in the Additional file 1. (4) Providing systematic analysis and comparison. It should be noted that not just one property affects the behavior of drugs in body. Usually we are looking for molecules that possess relatively good performance through every stage of ADME/T. ADMETlab allows users to evaluate most aspects of ADME/T process of one specific molecule, which gives users a full impression and leads to constructive suggestions of molecular optimization. (4) Supporting diverse similarity searching approaches. (5) Supporting batch computation. Calculating the properties for a single molecule is of little use for a chem- or bio-informatician who is dealing with ample data especially in virtual screening. ADMETlab supports the batch computation by uploading files. (6) Providing a convenient user-friendly interface. The rich prompts and robust verification systems in ADMETlab ensure a good user experience.

In order to give a more clear comparison we have listed all related web tools as possible as we know in Table 6. In the table we described their advantages/shortcomings and compared them with ADMETlab: (1) The “Similarity searching”, “Druglikeness model” and “Suggestion” functionalities are unique features of ADMETlab. (2) It seems that some tools are similar with ADMElab. There is no doubt that all of them contribute to ADMET properties prediction; however, they are quite different from ADMETlab both in methods and functionalities. Take admetSAR for example, the admetSAR built 22 classification models and 5 regression models with SVM methods, while ADMETlab systematically compared different methods (SVM, RF, NB, RP, PLS, DT) to get a proper method for each endpoint. In admetSAR, all compounds were represented using MACCS keys while ADMETlab systematically compared different descriptors and fingerprints (11 descriptor groups and 5 kinds of fingerprints) to get a more proper representation. It should be noted that the regression models based on SVM and MACCS keys are usually not very reliable in predicting continuous endpoints such as logS, logD, Caco-2 etc. Besides, ADMET combined larger datasets for most of the endpoints which represented broader chemical space. Moreover, ADMETlab provided batch computation which enables to screen libraries for qualified molecules. Another example is SwissADME, and it calculates 19 endpoints; however, it doesn’t calculate five kinds of CYP450 substrates, bioavailability, Clearance, T1/2, VD, Pgp-inhibitor, Caco-2, HIA, PPB and any toxicity endpoints. So, ADMETlab is very different from these tools and can be used as a new systematic ADMET evaluation platform owing to these unique features.

**Table 6:** Web tools related with ADMET prediction

Tools	Availability	Batch computation	Endpoints	Database	Druglikeness rules	Druglikeness model	Systematic evaluation	Suggestions
ADMETlab	Free	Yes	Number: 31 Contents: B, A, D, M, E, T*	Yes (288,967 entries; 5 similarity searching strategies)	Yes (5 rules)	Yes	Yes	Yes
lazar [41]	Free	No	Number: 3 Contents: T: Acute toxicity; BBB; Carcinogenicity	No	No	No	No	No
admetSAR [42]	Free	No	Number: 27 Contents: B, A, D, M, E, T	Yes (210,000 entries)	No	No	Yes	No
PreADMET [43]	Free or commercial	No	Number: 19 Contents: B, A, D, M, T	No	Yes	No	No	No
FAF-Drugs4 [44]	Free	Yes	Mainly filtering compounds by their descriptors and basic properties	No	Yes	No	No	No
pkCSM [12]	Free	Yes	Number: 30 Contents: B, A, D, M, E, T	No	No	No	Yes	No
SwissADME [45]	Free	Yes	Number: 19 Contents: B, A, D, M	No	Yes	No	Yes	No
VCCLAB [46]	Free	Yes	Number: 14 Contents: B (Different LogP, LogS and pKa from different theories)	No	No	No	No	No
Molinspiration [47]	Free	No	5 bioactivities, miLogP and 8 molecular descriptors	No	No	No	No	No
vNN-ADMET [48]	Registration required	No	Number: 14 Contents: A, D, M, T	No	No	No	No	No

*The “B, A, D, M, E, T” refers the contents in the “Documentation” section of our website. A tool that marked “A” means it covers some endpoints of class “A”, not all endpoints of class “A”

## Conclusion

ADMETlab provides a user-friendly, freely available web platform for systematic ADMET evaluation of chemicals based on a comprehensively collected database consisting of 288,967 entries. In this study, a series of well-performed prediction models were constructed based on different representation patterns and different modeling methods. With the assessment results, users can give an overall understanding of ADMET space, realize virtual screening or filtering and even obtain some hints about structure optimization. Additionally, some high-quality ADMET-related datasets are provided as benchmark datasets to improve the ADMET prediction. In the future, we will continue to improve the server as follows: (1) More practical models for new ADMET properties should be added, such as cytotoxicity and renal toxicity models. (2) Some hard-to-predict models should be further optimized, such as CL and T1/2 models. (3) The database should be updated regularly. (4) Integrated analysis based on ADMET profiles should be added to perform ADMET space analysis. In conclusion, we believe that this web platform will hopefully facilitate the drug discovery process by enabling the early evaluation, rapid ADMET virtual screening or filtering and prioritization of chemical structures.

## Additional file

**Additional file 1.** The detailed modeling process and results of the ADMET properties.

## References

[1] Mullard A (2014). 2013 FDA drug approvals. Nat Rev Drug Discov..

[2] Mullard A (2017). 2016 FDA drug approvals. Nat Rev Drug Discov..

[3] Fordyce CB, Roe MT, Ahmad T, Libby P, Borer JS, Hiatt WR (2015). Cardiovascular drug development: is it dead or just hibernating?. J Am Coll Cardiol.

[4] Cheng F, Li W, Liu G, Tang Y (2013). In silico ADMET prediction: recent advances, current challenges and future trends. Curr Top Med Chem.

[5] Wang Y, Xing J, Xu Y, Zhou N, Peng J, Xiong Z (2015). In silico ADME/T modelling for rational drug design. Q Rev Biophys.

[6] Wishart DS (2007). Improving early drug discovery through ADME modelling: an overview. Drugs R&D.

[7] Rosales-Hernandez MC, Correa-Basurto J (2015). The importance of employing computational resources for the automation of drug discovery. Expert Opin Drug Discov.

[8] Hou T (2015). Theme title: in silico ADMET predictions in pharmaceutical research. Adv Drug Deliver Rev..

[9] Tao L, Zhang P, Qin C, Chen SY, Zhang C, Chen Z (2015). Recent progresses in the exploration of machine learning methods as in silico ADME prediction tools. Adv Drug Deliver Rev..

[10] Wang N, Huang C, Dong J, Yao Z, Zhu M, Deng Z (2017). Predicting human intestinal absorption with modified random forest approach: a comprehensive evaluation of molecular representation, unbalanced data, and applicability domain issues. RSC Adv..

[11] Wang NN, Dong J, Deng YH, Zhu MF, Wen M, Yao ZJ (2016). ADME properties evaluation in drug discovery: prediction of Caco-2 cell permeability using a combination of NSGA-II and boosting. J Chem Inf Model.

[12] Pires DEV, Blundell TL, Ascher DB (2015). pkCSM: predicting small-molecule pharmacokinetic and toxicity properties using graph-based signatures. J Med Chem.

[13] Davies M, Dedman N, Hersey A, Papadatos G, Hall MD, Cucurull-Sanchez L (2015). ADME SARfari: comparative genomics of drug metabolizing systems. Bioinformatics.

[14] Dong J, Wang NN, Liu KY, Zhu MF, Yun YH, Zeng WB (2017). ChemBCPP: a freely available web server for calculating commonly used physicochemical properties. Chemometr Intell Lab Syst.

[15] Landrum. RDKit: open-source cheminformatics. Release 2014.03.1. 2010

[16] O’Boyle NM, Morley C, Hutchison GR (2008). Pybel: a Python wrapper for the OpenBabel cheminformatics toolkit. Chem Cent J.

[17] Cao D, Xu Q, Hu Q, Liang Y (2013). ChemoPy: freely available python package for computational biology and chemoinformatics. Bioinformatics.

[18] Dong J, Cao D, Miao H, Liu S, Deng B, Yun Y (2015). ChemDes: an integrated web-based platform for molecular descriptor and fingerprint computation. J Cheminform..

[19] Dong J, Yao ZJ, Wen M, Zhu MF, Wang NN, Miao HY (2016). BioTriangle: a web-accessible platform for generating various molecular representations for chemicals, proteins. DNAs/RNAs and their interactions. J Cheminform..

[20] Pedregosa F, Gramfort A, Michel V, Thirion B, Grisel O, Blondel M (2012). Scikit-learn: machine learning in Python. J Mach Learn Res..

[21] van der Walt S, Colbert SC, Varoquaux G (2011). The NumPy array: a structure for efficient numerical computation. Comput Sci Eng.

[22] Mckinney W (2017). Python for data analysis: data wrangling with Pandas, NumPy, and IPython.

[23] Gaulton A, Hersey A, Nowotka M, Bento AP, Chambers J, Mendez D (2017). The ChEMBL database in 2017. Nucleic Acids Res.

[24] EPA. https://www.epa.gov/. Accessed at 2018 Jan 15

[25] Wishart DS, Knox C, Guo AC, Shrivastava S, Hassanali M, Stothard P (2006). DrugBank: a comprehensive resource for in silico drug discovery and exploration. Nucleic Acids Res..

[26] Dong J, Yao ZJ, Zhu MF, Wang NN, Lu B, Chen AF (2017). ChemSAR: an online pipelining platform for molecular SAR modeling. J Cheminform.

[27] Breiman L (2001). Random forests. Mach Learn.

[28] Cao D, Yang Y, Zhao J, Yan J, Liu S, Hu Q (2012). Computer-aided prediction of toxicity with substructure pattern and random forest. J Chemometr.

[29] Cao D, Hu Q, Xu Q, Yang Y, Zhao J, Lu H (2011). In silico classification of human maximum recommended daily dose based on modified random forest and substructure fingerprint. Anal Chim Acta.

[30] Cao D, Dong J, Wang N, Wen M, Deng B, Zeng W (2015). In silico toxicity prediction of chemicals from EPA toxicity database by kernel fusion-based support vector machines. Chemometr Intell Lab..

[31] Strobl C, Malley J, Tutz G (2009). An introduction to recursive partitioning: rationale, application, and characteristics of classification and regression trees, bagging, and random forests. Psychol Methods.

[32] Wold S, Sjostrom M, Eriksson L (2001). PLS-regression: a basic tool of chemometrics. Chemometr Intell Lab..

[33] Cao D, Xu Q, Liang Y, Chen X, Li H (2010). Prediction of aqueous solubility of druglike organic compounds using partial least squares, back-propagation network and support vector machine. J Chemometr..

[34] Jiang W, Shen Y, Ding Y, Ye C, Zheng Y, Zhao P (2018). A naive Bayes algorithm for tissue origin diagnosis (TOD-Bayes) of synchronous multifocal tumors in the hepatobiliary and pancreatic system. Int J Cancer.

[35] Xia Y, Liu C, Da B, Xie F (2018). A novel heterogeneous ensemble credit scoring model based on bstacking approach. Expert Syst Appl.

[36] Lipinski CA, Lombardo F, Dominy BW, Feeney PJ (2001). Experimental and computational approaches to estimate solubility and permeability in drug discovery and development settings. Adv Drug Deliver Rev..

[37] Ghose AK, Viswanadhan VN, Wendoloski JJ. A knowledge based approach in designing combinatorial and medicinal chemistry libraries for drug discovery: 1. Qualitative and quantitative definitions of a drug like molecule. In: Abstracts of papers of the American Chemical Society, vol. 217, no. 1; 1999. p. U708. 10.1021/cc9800071 10746014

[38] Oprea TI (2000). Property distribution of drug-related chemical databases. J Comput Aid Mol Des..

[39] Veber DF, Johnson SR, Cheng HY, Smith BR, Ward KW, Kopple KD (2002). Molecular properties that influence the oral bioavailability of drug candidates. J Med Chem.

[40] Varma MVS, Obach RS, Rotter C, Miller HR, Chang G, Steyn SJ (2010). Physicochemical space for optimum oral bioavailability: contribution of human intestinal absorption and first-pass elimination. J Med Chem.

[41] Lazar, https://www.predictive-toxicology.org/. Accessed at 2018 Jan 15

[42] Cheng F, Li W, Zhou Y, Shen J, Wu Z, Liu G (2012). admetSAR: a comprehensive source and free tool for assessment of chemical ADMET properties. J Chem Inf Model.

[43] PreADMET. https://preadmet.bmdrc.kr/. Accessed at 2018 Jan 15

[44] Lagorce D, Bouslama L, Becot J, Miteva MA, Villoutreix BO (2017). FAF-Drugs4: free ADME-tox filtering computations for chemical biology and early stages drug discovery. Bioinformatics.

[45] Daina A, Michielin O, Zoete V (2017). SwissADME: a free web tool to evaluate pharmacokinetics, drug-likeness and medicinal chemistry friendliness of small molecules. Sci Rep UK.

[46] Tetko IV, Gasteiger J, Todeschini R, Mauri A, Livingstone D, Ertl P (2005). Virtual computational chemistry laboratory - design and description. J Comput Aid Mol Des..

[47] Molinspiration, http://www.molinspiration.com/. Accessed at 2018 Jan 15

[48] Schyman P, Liu R, Desai V (2017). vNN web server for ADMET predictions. Front Pharmacol.

