# Supplementary material for: ADMETlab: a platform for systematic ADMET evaluation based on a comprehensively collected ADMET database
Source: J Cheminform. 2018 Jun 26;10:29. doi: 10.1186/s13321-018-0283-x (PMC6020094; doi:10.1186/s13321-018-0283-x)
Supplement: Supplementary file 1 — Additional file 1. The detailed modeling process and results of the ADMET properties. [file 13321_2018_283_MOESM1_ESM.docx]

Contents

[1. Data collection 2](#_Toc503784399)

[1.1 Basic physicochemical property 2](#_Toc503784400)

[1.2 Absorption 3](#_Toc503784401)

[1.3 Distribution 5](#_Toc503784402)

[1.4 Metabolism 6](#_Toc503784403)

[1.5 Excretion 8](#_Toc503784404)

[1.6 Toxicity 8](#_Toc503784405)

[2. Results 11](#_Toc503784406)

[2.1 Basic physicochemical property 11](#_Toc503784407)

[2.2 Absorption 15](#_Toc503784408)

[2.3 Distribution 22](#_Toc503784409)

[2.4 Metabolism 27](#_Toc503784410)

[2.5 Excretion 34](#_Toc503784411)

[2.6 Toxicity 36](#_Toc503784412)

[3. Summary 39](#_Toc503784413)

[4. Reference 41](#_Toc503784414)

**Model selection and validation**

With the development of combinatorial chemistry and functional genomics, the number of new chemical entity has been increasing rapidly which is considered to be a good chance for drug discovery. However, available information suggests that the development of new drug still remains at a slow rate of 20% and the poor pharmacokinetics related properties (absorption, distribution, metabolism, excretion, ADME) and the drug toxicity account for half of the reported failures.[[1](#_ENREF_1), [2](#_ENREF_2)] Therefore, rapid and reliable estimation of these properties is certainly necessary for saving investment in the early stage of drug discovery. Although many individual models have been developed to predict some ADME/T properties, there are few open platforms for systemic ADME/T evaluation. In this study, we constructed a comprehensive platform named ADMET lab to accomplish a series of evaluation work necessary in the early stage of drug discovery. In the supporting information, we mainly provide the supplementary material about data collection, descriptor calculation and selection, modeling methods, performance evaluation and modeling results.

# Data collection

- 1. ****Basic physicochemical property****

**LogS:** The logarithm of aqueous solubility value. The first step in the drug absorption process is the disintegration of the tablet or capsule, followed by the dissolution of the active drug. Obviously, low solubility is detrimental to good and complete oral absorption, and so the early measurement of this property is of great importance in drug discovery.[[3](#_ENREF_3), [4](#_ENREF_4)] In this study, the solubility (LogS) data were obtained from two resources. One is Huuskonen’s work [[5](#_ENREF_5)] and the other is Delaney’s work and mainly consisted of low molecular weight organic compounds.[[6](#_ENREF_6)]

**LogD_7.4_:** The logarithm of the n-octanol/water distribution coefficients at pH=7.4. To exert a therapeutic effect, one drug must enter the blood circulation and then reach the site of action. Thus, an eligible drug usually needs to keep a balance between lipophilicity and hydrophilicity to dissolve in the body fluid and penetrate the biomembrane effectively.[[7-9](#_ENREF_7)] Therefore, it is important to estimate the n-octanol/water distribution coefficients at physiological pH (logD_7.4_) values for candidate compounds in the early stage of drug discovery. In this part, the dataset of logD_7.4_ was collected from our previous QSAR study and totally obtained 1131 compounds.[[10](#_ENREF_10)]

- 1. ****Absorption****

Absorption is the process that a drug enters human circulatory system from its administration place which can be found in various epithelial cell membranes including oral cavity, stomach, intestinal and so on. For an oral drug, the intestinal is the most important absorption site and consequently the human intestinal absorption of an oral drug is the essential prerequisite for its apparent efficacy. There are a lot of factors that influence the absorption of a drug at different degrees and they can be classified into three categories: physiological factors such as digestive system and circulatory system factors; physicochemical factors such as dissociation degree and liposolubility; dosage form factors such as the disintegration and dissolution of a drug. In this part, we studied 6 absorption-related endpoints and the data collection for them are described as follows.

**Caco-2 cell permeability:** Before an oral drug reaches the systemic circulation, it must pass through intestinal cell membranes via passive diffusion, carrier-mediated uptake or active transport processes. The human colon adenocarcinoma cell lines (Caco-2), as an alternative approach for the human intestinal epithelium, has been commonly used to estimate *in vivo* drug permeability due to their morphological and functional similarities.[[11-13](#_ENREF_11)] Thus, Caco-2 cell permeability has also been an important index for an eligible candidate drug compound. In this study, the dataset of Caco-2 cell permeability was also collected from a QSAR study carried out by our group and it contains 1182 compounds in total.[[14](#_ENREF_14)]

**Pgp-inhibitor:** The inhibitor of P-glycoprotein. The P-glycoprotein, also known as MDR1 or 2 ABCB1, is a membrane protein member of the ATP-binding cassette (ABC) transporters superfamily. Together with hERG channel and CYP3A4, it is probably the most widely studied antitarget. In fact, Pgp is probably the most promiscuous efflux transporter, since it recognizes a number of structurally different and apparently unrelated xenobiotics; notably, many of them are also CYP3A4 substrates.[[15](#_ENREF_15)] Consequently, the P-glycoprotein plays an important role not only in the absorption process, but also in other pharmacokinetic processes such as distribution, metabolism and excretion.[[16](#_ENREF_16), [17](#_ENREF_17)] In this study, Pgp-inhibitor data were obtained from two resources. One contains 1273 compounds were collected from Chen et al, including 797 Pgp inhibitors and 476 Pgp non-inhibitors.[[18](#_ENREF_18)] The other contains 1275 compounds were collected from Broccatelli et al., including 666 Pgp inhibitors and 609 Pgp non-inhibitors.[[15](#_ENREF_15)]

**Pgp-substrate:** The substrate of P-glycoprotein. As described in the Pgp-inhibitor section, the p-glycoprotein plays an important role in the ADME process for a drug compound and similar to the Pgp inhibitors, the estimation of Pgp substrates are also of high importance in the early stage of drug discovery. Pgp-substrate data were obtained from two resources. One dataset which contains 332 compounds were collected from Wang et al. and it includes 127 Pgp substrates and 205 Pgp non-substrates.[[19](#_ENREF_19)] One which contains 933 compounds were collected from Hou et al. and it includes 448 Pgp substrates and 485 Pgp non-substrates.[[20](#_ENREF_20)]

**HIA:** The human intestinal absorption. As described above, the human intestinal absorption of an oral drug is the essential prerequisite for its apparent efficacy. What’s more, the close relationship between oral bioavailability and intestinal absorption has also been proven and HIA can be seen an alternative indicator for oral bioavailability to some extent.[[21](#_ENREF_21)] In our study, the HIA dataset was collected from Hou’s work which contains 578 compounds and our study.[[22](#_ENREF_22), [23](#_ENREF_23)] To build a classification model, the positive and negative compounds were defined. If a compound with a HIA% less than 30%, it is labeled as negative; otherwise it is labeled as positive.

**F:** The human oral bioavailability. For any drug administrated by the oral route, oral bioavailability is undoubtedly one of the most important pharmacokinetic parameters because it is the indicator of the eﬃciency of the drug delivery to the systemic circulation. In this study, the human oral bioavailability dataset was obtained from Hou’s work.[[24](#_ENREF_24)] This dataset contains 1013 molecules. The range of bioavailability value is 0-100. Two thresholds (20% and 30%) were applied to split all the compounds into positive and negative compounds.[[25](#_ENREF_25)] If the threshold is 20%, the positive category contains 759 molecules (including bioavailability value equal to 20%) and the negative category contains 254 molecules. If the threshold is 30%, the positive category contains 672 molecules (including bioavailability value equal to 30%) and the negative category contains 341 molecules.

- 1. ****Distribution****

In general, the distribution of a drug is a transport process between the blood and tissues. After a drug was absorbed into blood from its administration place, the circulatory system will act as a transporter to deliver the drug to its target organ, target tissue and target site. As to the influence factors for distribution, there are mainly the physicochemical properties of the drug such as the structural characters and lipophicity of the drug and the physiological characters of human body such as the plasma protein binding, blood flow and the vascular permeability. These aforementioned factors can lead to the distribution difference of various drugs and directly influence the drug efficacy and drug safety. In this part, we studied 3 distribution-related endpoints and the data collection for them are described as follows.

**PPB:** The plasma protein binding. As we all know, one of the major mechanisms of drug uptake and distribution is through PPB, thus the binding of a drug to proteins in plasma has a strong influence on its pharmacodynamic behavior. On the one hand, PPB can directly influence the oral bioavailability because the free concentration of the drug is at stake when a drug binds to serum proteins in this process. On the other hand, the protein-drug complex can serve as a depot. Thus, it is necessary to evaluate it in the early stage in drug development. In this part, the PPB data was collected from recent literatures and DrugBank database (<http://www.drugbank.ca>) and totally 1822 compounds.[[26-29](#_ENREF_26)]

**VD**: The volume of distribution. The VD is a theoretical concept that connects the administered dose with the actual initial concentration present in the circulation and it is an important parameter to describe the *in vivo* distribution for drugs. In practical, we can speculate the distribution characters for an unknown compound according to its VD value, such as its condition binding to plasma protein, its distribution amount in body fluid and its uptake amount in tissues. Therefore, the VD is an essential index to be measured in the early stage of drug discovery. In this study, the data set was collected from Obach’s work which contains 544 compounds.

**BBB:** The blood brain barrier. The BBB is an important pharmacokinetic property of a drug is its ability or inability to penetrate the blood-brain barrier. BBB penetration is important for drugs that target receptors in the brain. Examples of these drugs are antipsychotics, antiepileptics, and antidepressants. For drugs not directed at targets in the brain, BBB penetration is undesirable as it would lead to unwanted CNS-related side effects.[[30](#_ENREF_30), [31](#_ENREF_31)] In this study, BBB data were obtained from two resources. One is Li’s work which contains 415 compounds.[[32](#_ENREF_32)] The other is Shen’s work which contains 1840 compounds.[[33](#_ENREF_33)]

- 1. ****Metabolism****

Metabolism is a signature of living systems, and enables organisms to create a viable environment within which to perform the complex biochemical transformations that maintain homeostasis. For about 75% of all drugs, metabolism is one of the major clearance pathways. The metabolic system has evolved as the main line of defence against foreign, hazardous substances, by transforming them into readily excretable metabolites.[[34](#_ENREF_34)] Metabolic systems are highly complex and adaptable. For this process, a plethora of diverse enzyme families are involved and they can commonly be classified to two categories: the microsomal enzyme such as cytochrome P450 (CYP) enzymes important for most drugs and the non-microsomal enzyme important for few drugs. Therefore, the recognition of the CYP 450 enzyme substrate or inhibitor for a molecule is of high importance in the drug development process. In this study, we studied 5 most popular metabolism-related insoforms: CYP1A2-inhibitor, CYP1A2-substrate CYP3A4-inhibitor, CYP3A4-substrate, CYP2C9-inhibitor, CYP2C9-substrate, CYP2C19-inhibitor, CYP2C19-subatrate, CYP2D6-inhibitor, CYP2D6-substrate. Their detailed information and data collection were as follows.[[35](#_ENREF_35)]

**CYP inhibitor:** the inhibitor of CYP1A2, 3A4, 2C19, 2C9 and 2D6 were obtained from the PubChem BioAssay database, AID:1851, a quantitative high throughput screening with in vitro bioluminescent assay against five major isoforms of cytochrome P450.[[36](#_ENREF_36)] The prepared dataset was downloaded from Rostkowski’s work. In Rostkowski’s work, the inorganic compounds, salts and mixtures, as well as entries classified as inconclusive were excluded from the dataset. For each of the five isoforms, 3000 compounds were extracted from the corresponding dataset to use as a test set, while the remaining compounds were used as a training set.[[37](#_ENREF_37)]

**CYP2C9-substrate:** the original data is from two resources. One is Tang’s work which contains 530 non-substrates and 142 substrates.[[38](#_ENREF_38)] The other is Hou’s work which contains 226 substrates.[[39](#_ENREF_39)] The 75 duplicate molecules of substrate were removed. In addition, there are 24 molecules which belong to substrate and non-substrate class. These molecules were then manually checked by retrieve them on DrugBank. Among them, 8 of 24 are substrates. 16 of 24 could not distinguish which class it belongs and were removed.

**CYP2D6-substrate:** the original data comes from two resources. One is Tang’s work which contains 480 non-substrates and 191 substrates.[[38](#_ENREF_38)] The other one is Zaretzki’s work which contains 270 substrates. The 75 duplicate molecules of substrate were removed.[[39](#_ENREF_39)] However, there are 16 molecules which belong to substrate and non-substrate class. These molecules were then manually checked by retrieve them on DrugBank. Among them, 4 of 16 are actually substrates. 12 molecules could not distinguish which class it belongs and were removed.

As to CYP1A2, CYP3A4 and CYP2C19 substrate, the datasets were collected from the PubChem BioAssay database, AID:1851, a quantitative high throughput screening with in vitro bioluminescent assay against five major isoforms of cytochrome P450.[[36](#_ENREF_36)] The inorganic compounds, salts and mixtures, as well as entries classified as inconclusive were excluded from the dataset.

- 1. ****Excretion****

For a drug compound, it will generally undergo the absorption process, distribution process, metabolism process and finally the excretion process after it entering into the human body. Excretion is an elimination process for *in vivo* drugs or their metabolites just as its name implies. The excretion properties of a molecule can influence the drug efficiency and corresponding drug side effects. In this part, we studied two important excretion-related endpoints and their description and data collection were described below in detail.

**CL:** The clearance of a drug. Clearance is an important pharmacokinetic parameter that defines, together with the volume of distribution, the half-life, and thus the frequency of dosing of a drug.[[3](#_ENREF_3)] The data set was collected from Obach’s work.[[40](#_ENREF_40)]

**T_1/2_:** The half-life of a drug. T_1/2_ is a hybrid concept that involves clearance and volume of distribution, and it is arguably more appropriate to have reliable estimates of these two properties instead.[[3](#_ENREF_3)] The data set was also collected from Obach’s work.[[40](#_ENREF_40)]

- 1. ****Toxicity****

**hERG:** The human ether-a-go-go related gene. The During cardiac depolarization and repolarization, a voltage-gated potassium channel encoded by hERG plays a major role in the regulation of the exchange of cardiac action potential and resting potential. The hERG blockade may cause long QT syndrome (LQTS), arrhythmia, and Torsade de Pointes (TdP), which lead to palpitations, fainting, or even sudden death.[[41](#_ENREF_41), [42](#_ENREF_42)] Therefore, assessment of hERG-related cardiotoxicity has become an important step in the drug design/discovery pipeline. In this study, we collected 655 hERG blocker from Hou’s study published in 2016.[[43](#_ENREF_43)]

**H-HT:** The human hepatotoxicity. Drug induced liver injury is of great concern for patient safety and a major cause for drug withdrawal from the market. Adverse hepatic effects in clinical trials often lead to a late and costly termination of drug development programs. Thus, the early identification of a hepatotoxic potential is of great importance to all stakeholders.[[44](#_ENREF_44), [45](#_ENREF_45)] In this study, we collected a human hepatotoxicity dataset from Mulliner’s study published in 2016 and this dataset contains 2171 compounds.[[46](#_ENREF_46)]

**Ames:** The Ames test for mutagenicity. As we all know, the mutagenic effect has a close relationship with the carcinogenicity. Nowadays, the most widely used assay for testing the mutagenicity of compounds is the Ames experiment which was invented by a professor named Ames.[[47](#_ENREF_47), [48](#_ENREF_48)] Considering the low interlaboratory reproducibility rate, it is really necessary to develop a good model for mutagenicity prediction instead of in vitro tests.[[49](#_ENREF_49)] 7619 compounds were collected in this study and they were from Tang’s study published in 2012.[[50](#_ENREF_50)]

**Skin sensitivity:** Skin sensitivity is an important toxicology endpoint of chemical hazard determination and safety assessment. The biological identification of skin sensitivity can be determined by a variety of biological experiments, such as DPRA/PPRA, KeratinoSens/LuSens, h-CLAT and LLNA experiments. In addition to the activity prediction study of different datasets and different methods, Chia-Chi Wang has recently developed a comprehensive database: SkinSensDB, containing 710 active data entries from different experiments. Here, we collected 407 compounds from Vinicius M.Alves’s publication aimed to LLNA experiment and 404 compounds were finally prepared to construct the prediction model.[[51](#_ENREF_51)]

**LD50 of acute toxicity**: The rat oral acute toxicity. Determination of acute toxicity in mammals (e.g. rats or mice) is one of the most important tasks for the safety evaluation of drug candidates. Because in vivo assays for oral acute toxicity in mammals are time-consuming and costly, there is thus an urgent need to develop in silico prediction models of oral acute toxicity. The related data were obtained from EPA database and 7397 chemicals were prepared for modeling after removing duplicates and missing values.[[52](#_ENREF_52)]

**DILI:** Drug-induced liver injury (DILI) has become the most common safety problem of drug withdrawal from the market over the past 50 years. Here DILI dataset were collected from YJ Xu’s publication which combines three published data sets and we finally obtained 475 chemicals for modeling study.[[53](#_ENREF_53)]

**FDAMDD**: The maximum recommended daily dose. This data source was obtained from Cao’s publication and we collected 803 small molecules to carry out the next model construction process.[[54](#_ENREF_54)]

For all the ADME/T related datasets, the following pretreatments were carried out to guarantee the quality and reliability of the data: 1) removing drug compounds that without explicit description for ADME/T properties 2) for the classification data, reserve only one entity if there are two or more same compounds 3) for the regression data, if there are two or more entries for a molecule, the arithmetic mean value of these values was adopted to reduce the random error when their fluctuations was in a reasonable limit, otherwise, this compound would be deleted. 4) Washing molecules by MOE software (disconnecting groups/metals in simple salts, keeping the largest molecular fragment and add explicit hydrogen). After that, a series of high-quality datasets were obtained. According to the Organization for Economic Co-operation and Development (OECD) principles, not only the internal validation is needed to verify the reliability and predictive ability of models, but also the external validation.[[14](#_ENREF_14)] Therefore, all the datasets were randomly divided into training set and test set by the Molecular Operating Environment software (MOE, version 2014). In this step, we set a threshold that 75% compounds were classified as training set and the remaining 25% compounds were classified as test set. The detailed information for these datasets can be seen in Table 1.

**Table 1.** The number of compounds of each property

| Category | Property | Total | Positive | Negative | Train | Test |
| --- | --- | --- | --- | --- | --- | --- |
| Basic physicochemical property | LogS | 5220 | - | - | 4116 | 1104 |
|  | LogD_7.4_ | 1031 | - | - | 773 | 258 |
|  | LogP |  |  |  |  |  |
| Absorption | Caco-2 | 1182 | - | - | 886 | 296 |
|  | Pgp-Inhibitor | 2297 | 1372 | 925 | 1723 | 574 |
|  | Pgp-Substrate | 1252 | 643 | 609 | 939 | 313 |
|  | HIA | 970 | 818 | 152 | 728 | 242 |
|  | F (20%) | 1013 | 759 | 254 | 760 | 253 |
|  | F (30%) | 1013 | 672 | 341 | 760 | 253 |
| Distribution | PPB | 1822 | - | - | 1368 | 454 |
|  | VD | 544 | - | - | 408 | 136 |
|  | BBB | 2237 | 540 | 1697 | 1678 | 559 |
| Metabolism | CYP 1A2-Inhibitor | 12145 | 5713 | 6432 | 9145 | 3000 |
|  | CYP 1A2-Substrate | 396 | 198 | 198 | 297 | 99 |
|  | CYP 3A4-Inhibitor | 11893 | 5047 | 6846 | 8893 | 3000 |
|  | CYP 3A4-Substrate | 1020 | 510 | 510 | 765 | 255 |
|  | CYP 2C9-Inhibitor | 11720 | 3960 | 7760 | 8720 | 3000 |
|  | CYP 2C9-Substrate | 784 | 278 | 506 | 626 | 156 |
|  | CYP 2C19-Inhibitor | 12272 | 5670 | 6602 | 9272 | 3000 |
|  | CYP 2C19-Substrate | 312 | 156 | 156 | 234 | 78 |
|  | CYP 2D6-Inhibitor | 12726 | 2342 | 10384 | 9726 | 3000 |
|  | CYP 2D6-Substrate | 816 | 352 | 464 | 611 | 205 |
| Excretion | Clearance | 544 | - | - | 408 | 136 |
|  | T_1/2_ | 544 | - | - | 408 | 136 |
| Toxicity | hERG | 655 | 451 | 204 | 392 | 263 |
|  | H-HT | 2171 | 1435 | 736 | 1628 | 543 |
|  | Ames | 7619 | 4252 | 3367 | 5714 | 1905 |
|  | Skin sensitivity | 404 | 274 | 130 | 323 | 81 |
|  | Rat oral acute toxicity | 7397 |  |  | 5917 | 1480 |
|  | DILI | 475 | 236 | 239 | 380 | 95 |
|  | FDAMDD | 803 | 442 | 361 | 643 | 160 |

# Results

- 1. ****Basic physicochemical property****

**LogS:** As described before, four regression models for predicting logS were developed by RF, SVM, RP and PLS. The descriptors used in modeling process were listed in Table 3 and the statistic results for four models can be seen in Table 4. The plot of predicted logS versus experimental logS for the training set and the test set is shown in Figure 1. From the table 4 and Figure 1, we can see that the regression model using RF was the best one (Q^2^=0.860, R_T_^2^=0.979). Compared with the model published in 2013 by Maryam Salahinejad (R^2^=0.90, R_T_^2^=0.90), our model was a little better from the perspective of statistics.[[55](#_ENREF_55)] For this best model, the Williams plot was applied to define its application domain in Figure 2. As can be seen in this figure, the majority of compounds in the training and test set fall within the AD, indicating these compounds are most likely to be well predicted by the RF model.

**Table 3.** Selected descriptors in modeling process

| **Selected descriptors (40)** |
| --- |
| MATSm2, TIAC, GMTIV, IC1, naro, MATSm1, nsulph, Tpc, slogPVSA7, bcutp1, AWeight, Tnc, MRVSA9, bcutp3, IC0, AW, Hy, bcutv10, MRVSA6, PC6, bcutm1, bcutm8, slogPVSA1, IDET, Chi10, TPSA, Weight, Rnc, naccr, bcutp5, Chiv4, bcutm2, Chiv1, bcutm3, Chiv9, ncarb, bcutm4, PEOEVSA5, LogP2, LogP |

**Table 4**. The statistic results of models built by RF, SVM, RP and PLS

| Method | Training  size | Test  size | mtry | $\mathbf{R}^{\mathbf{2}}$ | $\mathbf{Q}^{\mathbf{2}}$ | $\mathbf{R}_{\mathbf{T}}^{\mathbf{2}}$ | RMSE_F_ | RMSE_CV_ | RMSE_T_ |
| --- | --- | --- | --- | --- | --- | --- | --- | --- | --- |
| **RF** | **4116** | **1104** | **10** | **0.980** | **0.860** | **0.979** | **0.095** | **0.698** | **0.712** |
| SVM | 4116 | 1104 | - | 0.964 | 0.842 | 0.955 | 0.254 | 0.744 | 0.847 |
| RP | 4116 | 1104 | - | 0.956 | 0.838 | 0.921 | 0.370 | 0.813 | 0.895 |
| PLS | 4116 | 1104 | - | 0.906 | 0.801 | 0.913 | 0.621 | 0.836 | 0.823 |


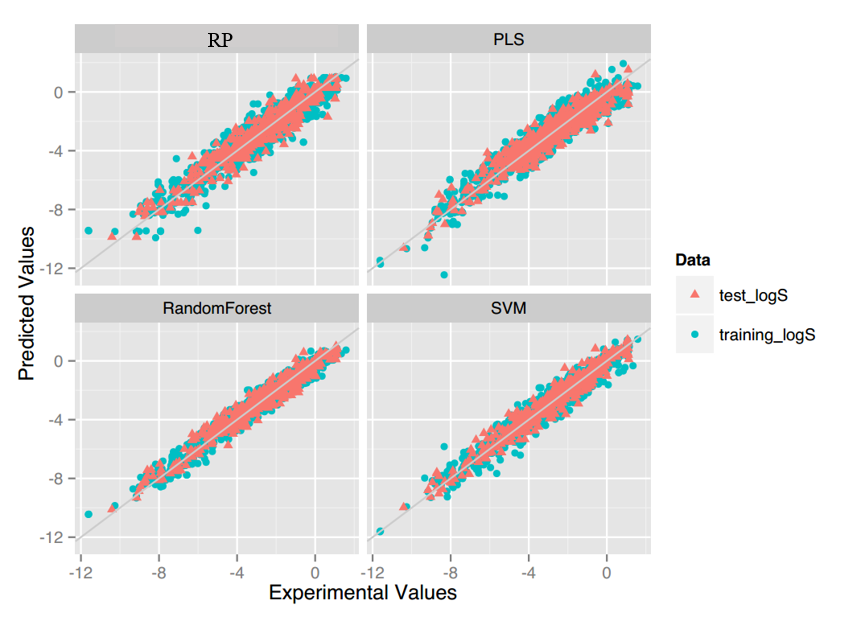


**Figure 1**. Plot of predicted logS versus experimental logS of models using four methods.


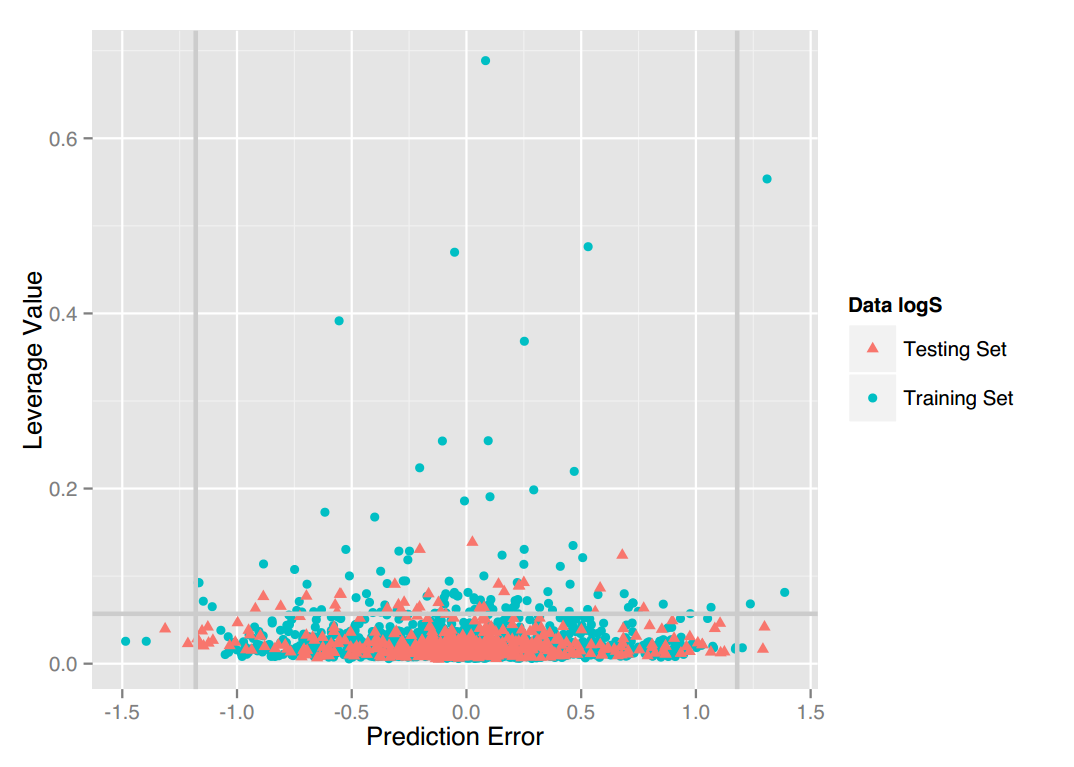


**Figure 2.** Williams plot of RF model.

**LogD_7.4_:** For this property, four regression models for predicting logD_7.4_ were developed by RF, SVM, RP and PLS. The descriptors used in modeling process were listed in Table 5 and the statistic results for four models can be seen in Table 6. The plot of predicted logD_7.4_ versus experimental logD_7.4_ for the training set and the test set is shown in Figure 3. From the table 6 and Figure 3, we can see that the regression model using RF was the best one (Q^2^=0.877, R_T_^2^=0.874). Up to now, the best model was built by us in 2015 (Q^2^=0.90, R_T_^2^=0.89), The two models have comparable performance.[[56](#_ENREF_56)] Some descriptors from our previous model are not supported in the server, so the results are not totally the same. For this best model, the Williams plot was applied to define its application domain in Figure 4. As can be seen in this figure, the majority of compounds in the training and test set fall within the AD, indicating these compounds are most likely to be well predicted by the RF model.

**Table 5.** Selected descriptors in modeling process

| **Selected descriptors (35)** |
| --- |
| MATSe5, PEOEVSA9, EstateVSA7, S13, EstateVSA0, Chiv4, S28, AW, QOmax, bcutp2, EstateVSA4, MATSe1, PC6, Hatov, S24, CIC0, QCmax, QCss, Geto, TPSA, Getov, bcutm11, CIC2, J, S34, PEOEVSA5, Hy, SPP, S36, S9, S16, MRVSA4, LogP2, QOmin, LogP |

**Table 6**. The statistic results of models built by RF, SVM, RP and PLS

| Method | Training  size | Test  size | mtry | $\mathbf{R}^{\mathbf{2}}$ | $\mathbf{Q}^{\mathbf{2}}$ | $\mathbf{R}_{\mathbf{T}}^{\mathbf{2}}$ | RMSE_F_ | RMSE_CV_ | RMSE_T_ |
| --- | --- | --- | --- | --- | --- | --- | --- | --- | --- |
| **RF** | **773** | **258** | **14** | **0.983** | **0.877** | **0.874** | **0.228** | **0.614** | **0.605** |
| SVM | 773 | 258 | - | 0.938 | 0.857 | 0.87 | 0.433 | 0.657 | 0.615 |
| RP | 773 | 258 | - | 0.912 | 0.783 | 0.745 | 0.515 | 0.88 | 0.793 |
| PLS | 773 | 258 | - | 0.756 | 0.728 | 0.768 | 0.86 | 0.909 | 0.82 |


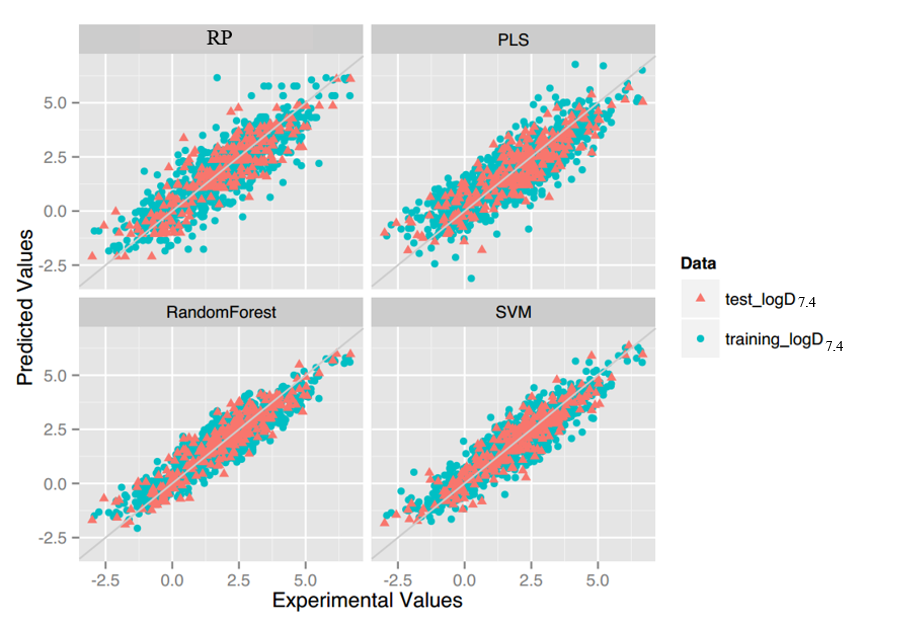


**Figure 3**. Plot of predicted values versus experimental values of models using four methods.


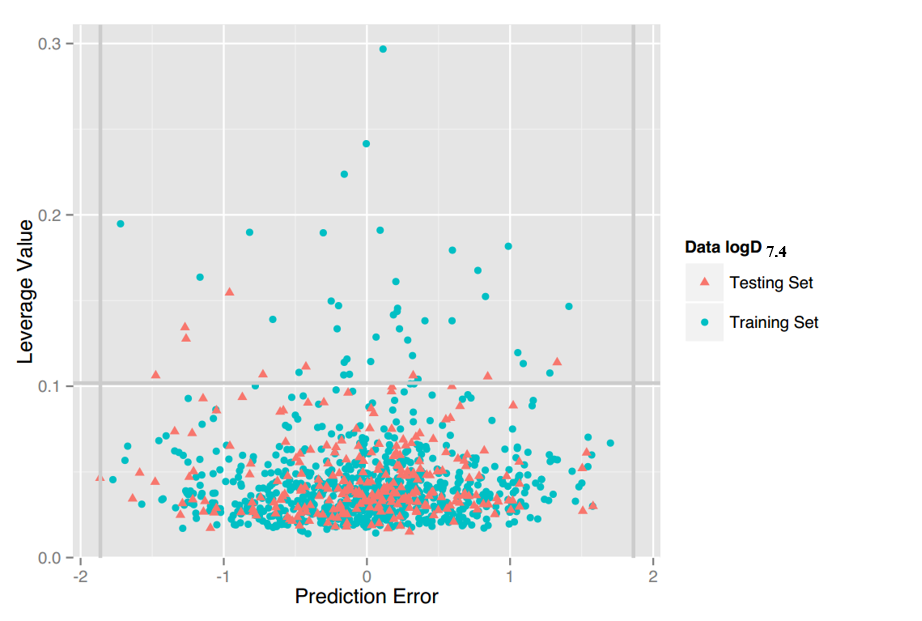


**Figure 4.** Williams plot of RF model.

- 1. ****Absorption****

**Caco-2:** For this property, four regression models for predicting Caco-2 were developed by RF, SVM, RP and PLS. The descriptors used in modeling process were listed in Table 7 and the statistic results for four models can be seen in Table 8. The plot of predicted Caco-2 versus experimental Caco-2 for the training set and the test set is shown in Figure 5. From the table 8 and Figure 5, we can see that the regression model using RF was the best one (Q^2^=0.845, R_T_^2^=0.824). Compared with the best model published in 2016 by us (Q^2^=0.83, R_T_^2^=0.81), this model was better from the perspective of statistics.[[57](#_ENREF_57)] For this best model, the Williams plot was applied to define its application domain in Figure 6. As can be seen in this figure, the majority of compounds in the training and test set fall within the AD, indicating these compounds are most likely to be well predicted by the RF model.

**Table 7.** Selected descriptors in modeling process

| **Selected descriptors (30)** |
| --- |
| ncarb, IC0, bcutp1, bcutv10, GMTIV, nsulph, CIC6, bcutm12, S34, bcutp8, slogPVSA2, QNmin, LogP2, bcutm1, EstateVSA9, slogPVSA1, Hatov, J, AW, S7, dchi0, MRVSA1, LogP, Tpc, PEOEVSA0, Tnc, S13, TPSA, QHss, ndonr |

**Table 8**. The statistic results of models built by RF, SVM, RP and PLS

| Method | Training  size | Test  size | mtry | $\mathbf{R}^{\mathbf{2}}$ | $\mathbf{Q}^{\mathbf{2}}$ | $\mathbf{R}_{\mathbf{T}}^{\mathbf{2}}$ | RMSE_F_ | RMSE_CV_ | RMSE_T_ |
| --- | --- | --- | --- | --- | --- | --- | --- | --- | --- |
| **RF** | **886** | **296** | **14** | **0.973** | **0.845** | **0.824** | **0.121** | **0.289** | **0.290** |
| SVM | 886 | 296 | - | 0.950 | 0.815 | 0.764 | 0.164 | 0.316 | 0.336 |
| RP | 886 | 296 | - | 0.884 | 0.683 | 0.657 | 0.250 | 0.414 | 0.405 |
| PLS | 886 | 296 | - | 0.690 | 0.657 | 0.627 | 0.409 | 0.430 | 0.422 |

**
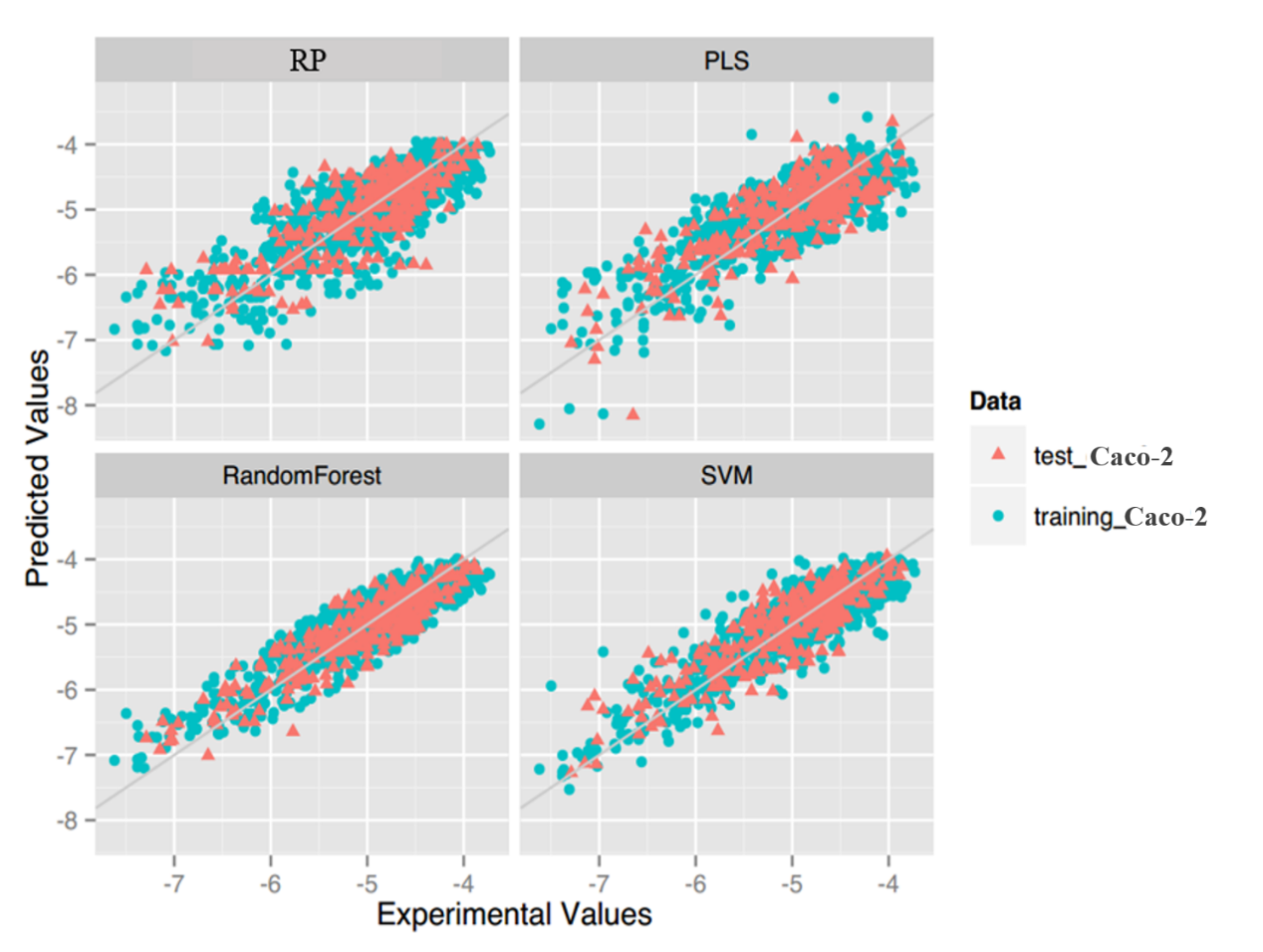
**

**Figure 5**. Plot of predicted values versus experimental values of models using four methods.

**
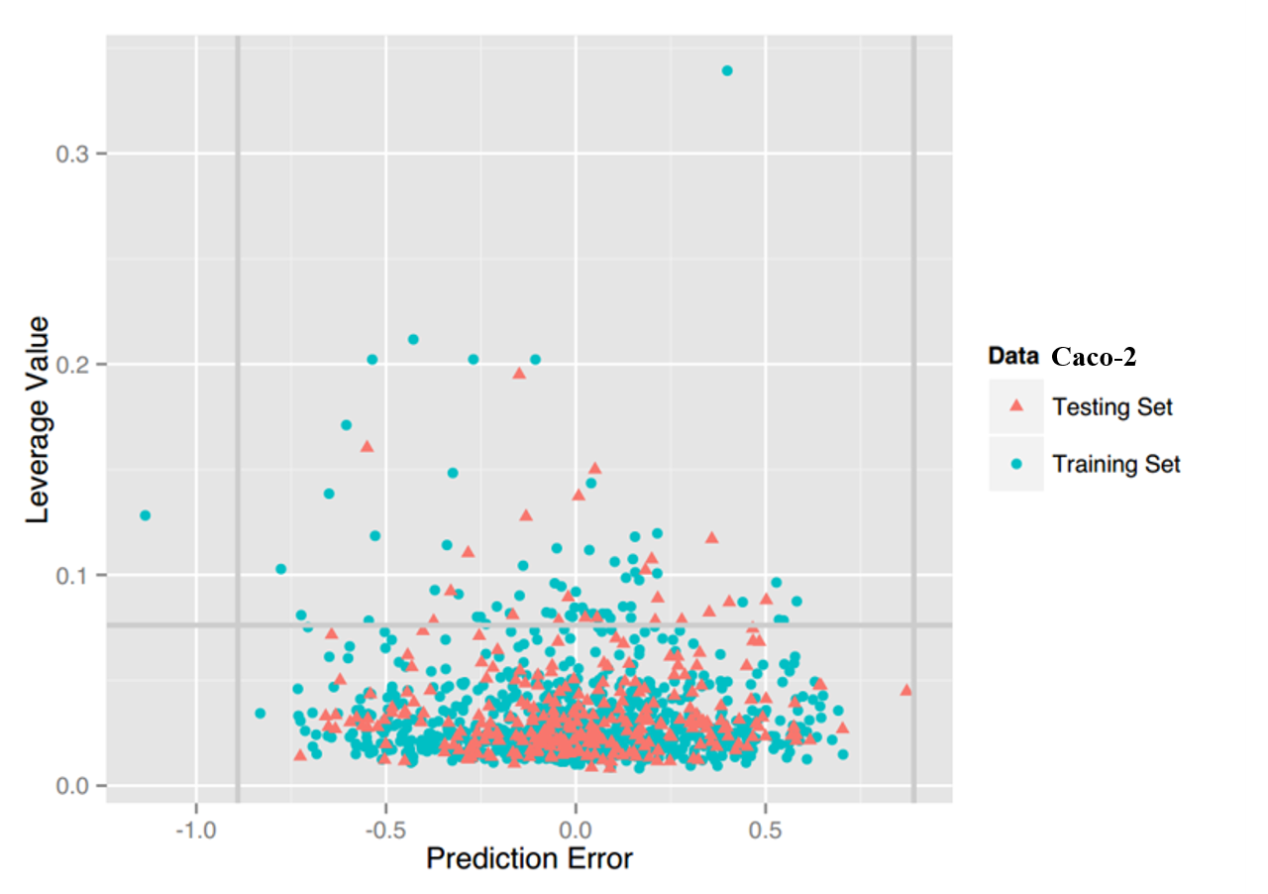
**

**Figure 6.** Williams plot of RF model.

**Pgp-Inhibitor:** For this property, 20 classification models were developed by four methods (RF, SVM, NB, DT) and five fingerprints (FP2, MACCS, ECFP2, ECFP4, ECFP6). The statistic results for these classification models can be seen in Table 9. From the table 9, we can see that the classification model based on SVM and ECFP4 was the best one with ACC=0.848 for the training set and ACC=0.838 for the test set. After searching for the existing models, we found that the best one was built by Lei Chen in 2011 (Tr: ACC=81.7, Te: ACC=81.2). Obviously, our obtained model was better than others and practical enough in future application.[[58](#_ENREF_58)]

**Table 9**. The statistic results of different classification models

| Method | fingerprint | Five folds cross validation | | | | External validation dataset | | | |
| --- | --- | --- | --- | --- | --- | --- | --- | --- | --- |
|  |  | Sensitivity | Specificity | Accuracy | AUC | Sensitivity | Specificity | Accuracy | AUC |
| DT | FP2 | 0.787 | 0.661 | 0.737 | 0.725 | 0.789 | 0.696 | 0.752 | 0.744 |
|  | MACCS | 0.817 | 0.710 | 0.774 | 0.766 | 0.810 | 0.731 | 0.779 | 0.777 |
|  | ECFP2 | 0.832 | 0.675 | 0.770 | 0.755 | 0.860 | 0.722 | 0.805 | 0.792 |
|  | ECFP4 | 0.802 | 0.680 | 0.754 | 0.743 | 0.845 | 0.714 | 0.793 | 0.780 |
|  | ECFP6 | 0.793 | 0.676 | 0.747 | 0.736 | 0.804 | 0.656 | 0.745 | 0.732 |
| BNB | FP2 | 0.712 | 0.574 | 0.657 | 0.652 | 0.716 | 0.542 | 0.647 | 0.641 |
|  | MACCS | 0.759 | 0.626 | 0.706 | 0.766 | 0.746 | 0.577 | 0.678 | 0.731 |
|  | ECFP2 | 0.827 | 0.707 | 0.779 | 0.858 | 0.822 | 0.718 | 0.780 | 0.852 |
|  | ECFP4 | 0.753 | 0.844 | 0.789 | 0.865 | 0.751 | 0.819 | 0.779 | 0.867 |
|  | ECFP6 | 0.723 | 0.859 | 0.777 | 0.866 | 0.711 | 0.877 | 0.777 | 0.870 |
| SVM | FP2^a^ | 0.859 | 0.747 | 0.814 | 0.892 | 0.863 | 0.771 | 0.826 | 0.897 |
|  | MACCS^b^ | 0.881 | 0.767 | 0.836 | 0.897 | 0.877 | 0.780 | 0.838 | 0.898 |
|  | ECFP2^c^ | 0.885 | 0.775 | 0.841 | 0.905 | 0.851 | 0.802 | 0.838 | 0.906 |
|  | **ECFP4^d^** | **0.887** | **0.789** | **0.848** | **0.908** | **0.863** | **0.802** | **0.838** | **0.913** |
|  | ECFP6^e^ | 0.890 | 0.804 | 0.856 | 0.907 | 0.824 | 0.860 | 0.845 | 0.912 |
| RF | FP2^f^ | 0.877 | 0.711 | 0.811 | 0.886 | 0.871 | 0.771 | 0.831 | 0.905 |
|  | MACCS^g^ | 0.880 | 0.761 | 0.833 | 0.899 | 0.901 | 0.767 | 0.847 | 0.916 |
|  | ECFP2^h^ | 0.877 | 0.766 | 0.833 | 0.901 | 0.886 | 0.806 | 0.854 | 0.918 |
|  | ECFP4^i^ | 0.865 | 0.779 | 0.830 | 0.899 | 0.883 | 0.802 | 0.851 | 0.917 |
|  | ECFP6^j^ | 0.873 | 0.770 | 0.832 | 0.897 | 0.874 | 0.789 | 0.840 | 0.912 |

a Coarse grid-search best: C = 2^3^, gamma = 2^-11^, finer grid-search best: C = 2^1.5^, gamma= 2^-9.75^

b Coarse grid-search best: C = 2^1^, gamma =2^-5^, finer grid-search best: C = 2^1^, gamma=2^-4.75^

c Coarse grid-search best: C = 2^1^, gamma = 2^-5^, finer grid-search best: C=2^1^, gamma=2^-4.25^

d Coarse grid-search best: C = 2^1^, gamma = 2^-7^, finer grid-search best: C = 2^1^, gamma = 2^-6.5^

e Coarse grid-search best: C = 2^0^, gamma = 2^-7^, finer grid-search best: C = 2^0.75^ , gamma = 2^-6.5^

f mtry = 1200

g mtry = 40

h mtry = 60

i mtry = 20

j mtry = 20

**Pgp-Substrate:** For this property, 20 classification models were developed by four methods (RF, SVM, NB, DT) and five fingerprints (FP2, MACCS, ECFP2, ECFP4, ECFP6). The statistic results for these classification models can be seen in Table 10. From the table 10, we can see that the classification model based on SVM and ECFP4 was the best one with ACC=0.824 for the training set and ACC=0.840 for the test set. Compared with the model published in 2014 (Tr: ACC=0.912, Te: ACC=0.835), our prediction model has a comparable and reasonable statistic result.[[20](#_ENREF_20)]

**Table 10**. The statistic results of different classification models

| Method | fingerprint | Five folds cross validation | | | | External validation dataset | | | |
| --- | --- | --- | --- | --- | --- | --- | --- | --- | --- |
|  |  | Sensitivity | Specificity | Accuracy | AUC | Sensitivity | Specificity | Accuracy | AUC |
| DT | FP2 | 0.689 | 0.589 | 0.640 | 0.639 | 0.683 | 0.609 | 0.647 | 0.648 |
|  | MACCS | 0.752 | 0.724 | 0.738 | 0.738 | 0.689 | 0.682 | 0.686 | 0.688 |
|  | ECFP2 | 0.777 | 0.735 | 0.756 | 0.756 | 0.689 | 0.742 | 0.715 | 0.716 |
|  | ECFP4 | 0.741 | 0.707 | 0.724 | 0.724 | 0.745 | 0.742 | 0.744 | 0.744 |
|  | ECFP6 | 0.731 | 0.681 | 0.706 | 0.705 | 0.714 | 0.722 | 0.718 | 0.718 |
| BNB | FP2 | 0.614 | 0.578 | 0.596 | 0.601 | 0.646 | 0.589 | 0.619 | 0.624 |
|  | MACCS | 0.783 | 0.713 | 0.749 | 0.795 | 0.727 | 0.728 | 0.728 | 0.820 |
|  | ECFP2 | 0.674 | 0.825 | 0.748 | 0.835 | 0.652 | 0.848 | 0.747 | 0.859 |
|  | ECFP4 | 0.651 | 0.875 | 0.761 | 0.843 | 0.596 | 0.894 | 0.740 | 0.844 |
|  | ECFP6 | 0.637 | 0.882 | 0.756 | 0.839 | 0.590 | 0.907 | 0.744 | 0.845 |
| SVM | FP2^a^ | 0.793 | 0.790 | 0.792 | 0.855 | 0.801 | 0.815 | 0.807 | 0.880 |
|  | MACCS^b^ | 0.791 | 0.827 | 0.809 | 0.881 | 0.839 | 0.868 | 0.853 | 0.932 |
|  | ECFP2^c^ | 0.827 | 0.821 | 0.824 | 0.896 | 0.795 | 0.841 | 0.817 | 0.907 |
|  | **ECFP4^d^** | **0.839** | **0.807** | **0.824** | **0.899** | **0.826** | **0.854** | **0.840** | **0.905** |
|  | ECFP6^e^ | 0.802 | 0.832 | 0.816 | 0.894 | 0.789 | 0.874 | 0.830 | 0.895 |
| RF | FP2^f^ | 0.701 | 0.823 | 0.761 | 0.833 | 0.764 | 0.821 | 0.792 | 0.861 |
|  | MACCS^g^ | 0.810 | 0.786 | 0.798 | 0.881 | 0.876 | 0.808 | 0.843 | 0.913 |
|  | ECFP2^h^ | 0.804 | 0.842 | 0.823 | 0.897 | 0.814 | 0.841 | 0.827 | 0.899 |
|  | ECFP4^i^ | 0.772 | 0.851 | 0.811 | 0.892 | 0.795 | 0.841 | 0.817 | 0.894 |
|  | ECFP6^j^ | 0.775 | 0.840 | 0.807 | 0.882 | 0.795 | 0.828 | 0.811 | 0.891 |

a Coarse grid-search best: C = 2^15^, gamma = 2^-9^, finer grid-search best: C = 2^15.25^, gamma= 2^-8.75^

b Coarse grid-search best: C = 2^1^, gamma =2^-3^, finer grid-search best: C = 2^0.5^, gamma=2^-3.5^

c Coarse grid-search best: C = 2^1^, gamma = 2^-5^, finer grid-search best: C=2^1.25^, gamma=2^-3.25^

d Coarse grid-search best: C = 2^7^, gamma = 2^-5^, finer grid-search best: C = 2^8.75^, gamma = 2^-5^

e Coarse grid-search best: C = 2^1^, gamma = 2^-7^, finer grid-search best: C = 2^1^, gamma = 2^-7^

f mtry = 150

g mtry = 20

h mtry = 10

i mtry = 10

j mtry = 10

**HIA:** For this property, 20 classification models were developed by four methods (RF, SVM, NB, DT) and five fingerprints (FP2, MACCS, ECFP2, ECFP4, ECFP6). The statistic results for these classification models can be seen in Table 11. From the table 11, we can see that the classification models based on SVM, NB and DT were unbalanced, and thus as described before, a new method based on RF was applied to obtain the balanced model. The best model based on RF and MACCS has an ACC=0.782 for the training set and ACC=0.773 for the test set. Compared with the recent model built by us (SE=0.877, SP=0.813), this new model has a comparable result.[[59](#_ENREF_59)] Some descriptors from our previous model are not supported in the server, so the results are not totally the same.

**Table 11**. The statistic results of different classification models

| Method | fingerprint | Five folds cross validation | | | | External validation dataset | | | |
| --- | --- | --- | --- | --- | --- | --- | --- | --- | --- |
|  |  | Sensitivity | Specificity | Accuracy | AUC | Sensitivity | Specificity | Accuracy | AUC |
| DT | FP2 | 0.759 | 0.561 | 0.733 | 0.660 | 0.784 | 0.553 | 0.768 | 0.769 |
|  | MACCS | 0.780 | 0.512 | 0.771 | 0.746 | 0.800 | 0.553 | 0.763 | 0.777 |
|  | ECFP2 | 0.780 | 0.503 | 0.770 | 0.741 | 0.792 | 0.553 | 0.766 | 0.773 |
|  | ECFP4 | 0.787 | 0.550 | 0.769 | 0.718 | 0.800 | 0.507 | 0.766 | 0.753 |
|  | ECFP6 | 0.760 | 0.567 | 0.748 | 0.714 | 0.792 | 0.507 | 0.749 | 0.749 |
| BNB | FP2 | 0.546 | 0.575 | 0.523 | 0.664 | 0.743 | 0.451 | 0.529 | 0.516 |
|  | MACCS | 0.699 | 0.596 | 0.685 | 0.618 | 0.778 | 0.567 | 0.661 | 0.717 |
|  | ECFP2 | 0.784 | 0.478 | 0.718 | 0.698 | 0.776 | 0.405 | 0.720 | 0.765 |
|  | ECFP4 | 0.773 | 0.584 | 0.722 | 0.716 | 0.767 | 0.434 | 0.724 | 0.763 |
|  | ECFP6 | 0.777 | 0.558 | 0.722 | 0.724 | 0.767 | 0.498 | 0.727 | 0.758 |
| SVM | FP2^a^ | 0.796 | 0.526 | 0.761 | 0.785 | 0.800 | 0.460 | 0.779 | 0.799 |
|  | MACCS^b^ | 0.792 | 0.529 | 0.784 | 0.795 | 0.801 | 0.554 | 0.798 | 0.723 |
|  | ECFP2^c^ | 0.795 | 0.567 | 0.778 | 0.797 | 0.798 | 0.545 | 0.737 | 0.722 |
|  | ECFP4^d^ | 0.797 | 0.567 | 0.780 | 0.796 | 0.799 | 0.553 | 0.722 | 0.712 |
|  | ECFP6^e^ | 0.795 | 0.558 | 0.777 | 0.794 | 0.801 | 0.553 | 0.793 | 0.798 |
| RF | FP2^f^ | 0.791 | 0.670 | 0.762 | 0.778 | 0.700 | 0.714 | 0.772 | 0.793 |
|  | **MACCS^g^** | **0.820** | **0.743** | **0.782** | **0.846** | **0.801** | **0.743** | **0.773** | **0.831** |
|  | ECFP2^h^ | 0.795 | 0.714 | 0.771 | 0.795 | 0.700 | 0.660 | 0.879 | 0.798 |
|  | ECFP4^i^ | 0.799 | 0.661 | 0.768 | 0.792 | 0.745 | 0.714 | 0.772 | 0.798 |
|  | ECFP6^j^ | 0.799 | 0.643 | 0.765 | 0.788 | 0.734 | 0.714 | 0.772 | 0.797 |

a Coarse grid-search best: C = 2^7^, gamma = 2^-9^, finer grid-search best: C = 2^8.5^, gamma=2^-7.25^

b Coarse grid-search best: C = 2^11^, gamma =2^-7^, finer grid-search best: C = 2^10.5^, gamma=2^-6^

c Coarse grid-search best: C = 2^13^, gamma = 2^-5^, finer grid-search best: C=2^13.75^, gamma=2^-4.5^

d Coarse grid-search best: C = 2^13^, gamma = 2^-5^, finer grid-search best: C = 2^13.25^, gamma=2^-6.25^

e Coarse grid-search best: C = 2^3^, gamma = 2^-7^, finer grid-search best: C = 2^4.25^ , gamma = 2^-8.5^

f mtry = 40

g mtry = 40

h mtry = 20

i mtry = 10

j mtry = 10

**F:** For this property, there were two thresholds (20% and 30%) for its classification. For each threshold, 20 classification models were developed by four methods (RF, SVM, NB, DT) and five fingerprints (FP2, MACCS, ECFP2, ECFP4, ECFP6). The statistic results for these classification models can be seen in Table 12 and Table 13. From the two tables, we can see that these models are unbalanced and thus some balanced models are built as described before. The classification model for F (20%) based on RF and MACCS was the best one with ACC=0.689 for the training set and ACC=0.671 for the test set. From the table 13, we can see that the classification model for F (30%) based on RF and ECFP6 was the best one with ACC=0.669 for the training set and ACC=0.667 for the test set. In 2012, Ahmed and Ramakrishnan developed a good classifier achieving a classification accuracy of 71% for the training set based on 969 compounds. Compared with it, our prediction model was further validated and had a comparable result.[[60](#_ENREF_60)]

**Table 12**. The statistic results of different classification models for F (20%)

| Method | fingerprint | Five folds cross validation | | | | External validation dataset | | | |
| --- | --- | --- | --- | --- | --- | --- | --- | --- | --- |
|  |  | Sensitivity | Specificity | Accuracy | AUC | Sensitivity | Specificity | Accuracy | AUC |
| DT | FP2 | 0.739 | 0.423 | 0.660 | 0.581 | 0.775 | 0.400 | 0.679 | 0.588 |
|  | MACCS | 0.808 | 0.455 | 0.720 | 0.631 | 0.813 | 0.462 | 0.722 | 0.637 |
|  | ECFP2 | 0.825 | 0.429 | 0.726 | 0.627 | 0.845 | 0.369 | 0.722 | 0.607 |
|  | ECFP4 | 0.762 | 0.423 | 0.677 | 0.593 | 0.759 | 0.462 | 0.683 | 0.610 |
|  | ECFP6 | 0.771 | 0.434 | 0.687 | 0.602 | 0.722 | 0.431 | 0.647 | 0.576 |
| BNB | FP2 | 0.586 | 0.566 | 0.581 | 0.578 | 0.642 | 0.569 | 0.623 | 0.593 |
|  | MACCS | 0.686 | 0.587 | 0.661 | 0.707 | 0.775 | 0.554 | 0.718 | 0.755 |
|  | ECFP2 | 0.935 | 0.296 | 0.775 | 0.702 | 0.925 | 0.308 | 0.766 | 0.771 |
|  | ECFP4 | 0.894 | 0.354 | 0.759 | 0.715 | 0.909 | 0.400 | 0.778 | 0.746 |
|  | ECFP6 | 0.882 | 0.370 | 0.754 | 0.698 | 0.898 | 0.446 | 0.782 | 0.722 |
| SVM | FP2^a^ | 0.912 | 0.280 | 0.754 | 0.693 | 0.909 | 0.431 | 0.786 | 0.705 |
|  | MACCS^b^ | 0.907 | 0.450 | 0.792 | 0.749 | 0.904 | 0.431 | 0.782 | 0.727 |
|  | ECFP2^c^ | 0.945 | 0.275 | 0.778 | 0.768 | 0.920 | 0.400 | 0.786 | 0.747 |
|  | ECFP4^d^ | 0.963 | 0.212 | 0.775 | 0.774 | 0.930 | 0.415 | 0.708 | 0.768 |
|  | ECFP6^e^ | 0.972 | 0.127 | 0.761 | 0.763 | 0.957 | 0.292 | 0.786 | 0.782 |
| RF | FP2^f^ | 0.947 | 0.217 | 0.765 | 0.667 | 0.925 | 0.323 | 0.770 | 0.713 |
|  | MACCS^g^ | 0.940 | 0.291 | 0.778 | 0.754 | 0.925 | 0.369 | 0.782 | 0.794 |
|  | ECFP2^h^ | 0.951 | 0.265 | 0.779 | 0.753 | 0.963 | 0.323 | 0.798 | 0.759 |
|  | ECFP4^i^ | 0.966 | 0.190 | 0.772 | 0.742 | 0.973 | 0.292 | 0.798 | 0.771 |
|  | ECFP6^j^ | 0.977 | 0.101 | 0.758 | 0.739 | 0.984 | 0.215 | 0.786 | 0.769 |
|  | **MACCS** | **0.731** | **0.647** | **0.689** | **0.759** | **0.680** | **0.663** | **0.671** | **0.746** |

a Coarse grid-search best: C = 2^9^, gamma = 2^-9^, finer grid-search best: C = 2^9.5^, gamma=2^-8.5^

b Coarse grid-search best: C = 2^7^, gamma =2^-9^, finer grid-search best: C = 2^7.5^, gamma=2^-9^

c Coarse grid-search best: C = 2^3^, gamma = 2^-5^, finer grid-search best: C=2^1.25^, gamma=2^-3.75^

d Coarse grid-search best: C = 2^7^, gamma = 2^-5^, finer grid-search best: C = 2^8.5^, gamma=2^-4.75^

e Coarse grid-search best: C = 2^11^, gamma = 2^-5^, finer grid-search best: C = 2^10.25^ , gamma = 2^-5^

f mtry = 500

g mtry = 20

h mtry = 80

i mtry = 20

j mtry = 10

**Table 13**. The statistic results of different classification models for F (30%)

| Method | fingerprint | Five folds cross validation | | | | External validation dataset | | | |
| --- | --- | --- | --- | --- | --- | --- | --- | --- | --- |
|  |  | Sensitivity | Specificity | Accuracy | AUC | Sensitivity | Specificity | Accuracy | AUC |
| DT | FP2 | 0.689 | 0.522 | 0.634 | 0.606 | 0.722 | 0.544 | 0.659 | 0.633 |
|  | MACCS | 0.764 | 0.530 | 0.687 | 0.647 | 0.642 | 0.600 | 0.627 | 0.621 |
|  | ECFP2 | 0.778 | 0.510 | 0.689 | 0.644 | 0.698 | 0.556 | 0.647 | 0.627 |
|  | ECFP4 | 0.731 | 0.506 | 0.656 | 0.618 | 0.698 | 0.556 | 0.647 | 0.627 |
|  | ECFP6 | 0.713 | 0.546 | 0.657 | 0.629 | 0.698 | 0.533 | 0.639 | 0.615 |
| BNB | FP2 | 0.596 | 0.566 | 0.586 | 0.575 | 0.593 | 0.533 | 0.571 | 0.568 |
|  | MACCS | 0.663 | 0.594 | 0.640 | 0.685 | 0.704 | 0.567 | 0.655 | 0.676 |
|  | ECFP2 | 0.897 | 0.398 | 0.731 | 0.727 | 0.833 | 0.367 | 0.667 | 0.694 |
|  | ECFP4 | 0.846 | 0.466 | 0.720 | 0.739 | 0.827 | 0.400 | 0.675 | 0.685 |
|  | ECFP6 | 0.865 | 0.498 | 0.743 | 0.739 | 0.765 | 0.433 | 0.647 | 0.679 |
| SVM | FP2^a^ | 0.909 | 0.394 | 0.738 | 0.736 | 0.866 | 0.385 | 0.689 | 0.710 |
|  | MACCS^b^ | 0.917 | 0.386 | 0.741 | 0.752 | 0.870 | 0.390 | 0.692 | 0.712 |
|  | ECFP2^c^ | 0.885 | 0.494 | 0.755 | 0.782 | 0.872 | 0.394 | 0.695 | 0.699 |
|  | ECFP4^d^ | 0.919 | 0.486 | 0.775 | 0.788 | 0.874 | 0.400 | 0.702 | 0.718 |
|  | ECFP6^e^ | 0.927 | 0.402 | 0.753 | 0.790 | 0.877 | 0.400 | 0.706 | 0.720 |
| RF | FP2^f^ | 0.929 | 0.335 | 0.731 | 0.723 | 0.847 | 0.452 | 0.719 | 0.729 |
|  | MACCS^g^ | 0.869 | 0.458 | 0.733 | 0.764 | 0.858 | 0.478 | 0.722 | 0.738 |
|  | ECFP2^h^ | 0.927 | 0.402 | 0.753 | 0.786 | 0.877 | 0.400 | 0.706 | 0.720 |
|  | ECFP4^i^ | 0.947 | 0.371 | 0.755 | 0.781 | 0.889 | 0.322 | 0.687 | 0.721 |
|  | ECFP6^j^ | 0.949 | 0.339 | 0.746 | 0.786 | 0.907 | 0.311 | 0.694 | 0.729 |
|  | **ECFP6** | **0.743** | **0.605** | **0.669** | **0.715** | **0.751** | **0.601** | **0.667** | **0.718** |

a Coarse grid-search best: C = 2^1^, gamma = 2^-9^, finer grid-search best: C = 2^2.75^, gamma=2^-8^

b Coarse grid-search best: C = 2^1^, gamma =2^-3^, finer grid-search best: C = 2^1.5^, gamma=2^-3.25^

c Coarse grid-search best: C = 2^11^, gamma = 2^-3^, finer grid-search best: C=2^9.75^, gamma=2^-4^

d Coarse grid-search best: C = 2^3^, gamma = 2^-5^, finer grid-search best: C = 2^4.5^, gamma=2^-5.25^

e Coarse grid-search best: C = 2^11^, gamma = 2^-5^, finer grid-search best: C = 2^10.75^ , gamma = 2^-5.25^

f mtry = 60

g mtry = 40

h mtry = 40

i mtry = 20

j mtry = 10

- 1. ****Distribution****

**PPB:** For this property, four regression models for predicting PPB were developed by RF and different kinds of descriptors. The statistic results for three models can be seen in Table 14. The plot of predicted versus experimental values for the training set and the test set is shown in Figure 7. From the Table 14 and Figure 7, we can see that the regression model using RF and 2D descriptor was the best one (Q^2^=0.691, R_T_^2^=0.682). Compared with our recent work (Q^2^=0.750, R_T_^2^=0.787), the statistic result seems a little bit worse. [[61](#_ENREF_61)] Some descriptors from our previous model are not supported in the server, so the results are not totally the same. For this best model, the Williams plot was applied to define its application domain in Figure 8. As can be seen in this figure, the majority of compounds in the training and test set fall within the AD, indicating these compounds are most likely to be well predicted by the RF model.

**Table 14**. The statistic results of models built based on different descriptors

| **Descriptor** | **Training** | **Test** | $\mathbf{R}^{\mathbf{2}}$ | $\mathbf{Q}^{\mathbf{2}}$ | $\mathbf{R}_{\mathbf{T}}^{\mathbf{2}}$ | **RMSE_F_** | **RMSE_CV_** | **RMSE_T_** |
| --- | --- | --- | --- | --- | --- | --- | --- | --- |
| **2D** | **1368** | **454** | **0.954** | **0.691** | **0.682** | **7.124** | **18.443** | **18.044** |
| MACCS | 1368 | 454 | 0.943 | 0.589 | 0.632 | 7.965 | 21.327 | 19.632 |
| Estate | 1368 | 454 | 0.944 | 0.604 | 0.644 | 7.849 | 20.942 | 19.308 |

**
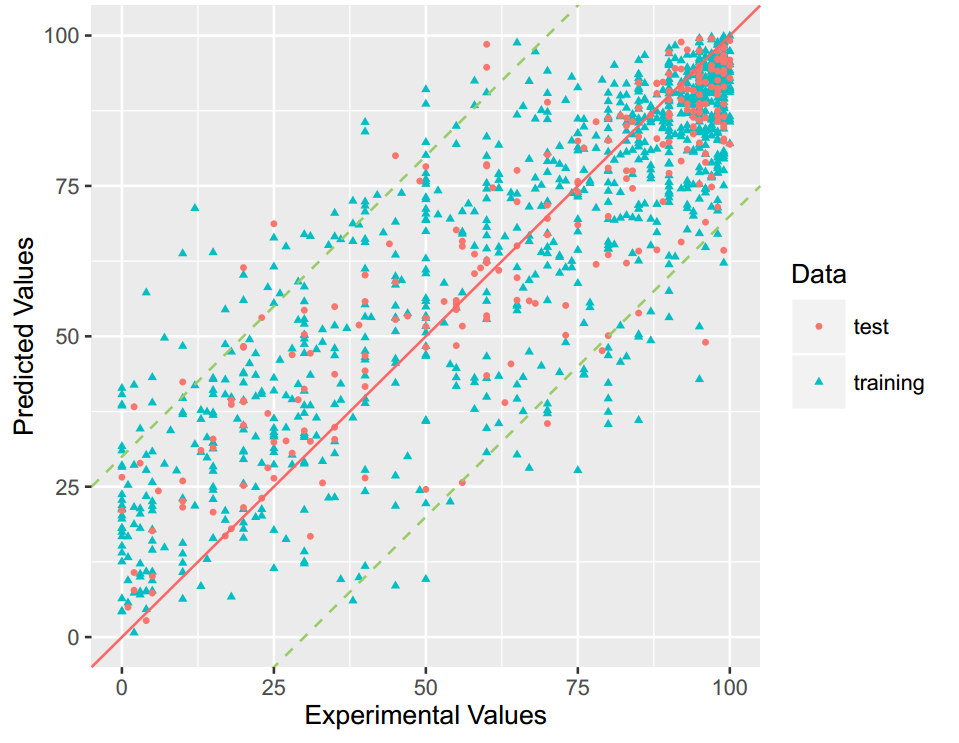
**

**Figure 7.** Plot of predicted values versus experimental values of models

**
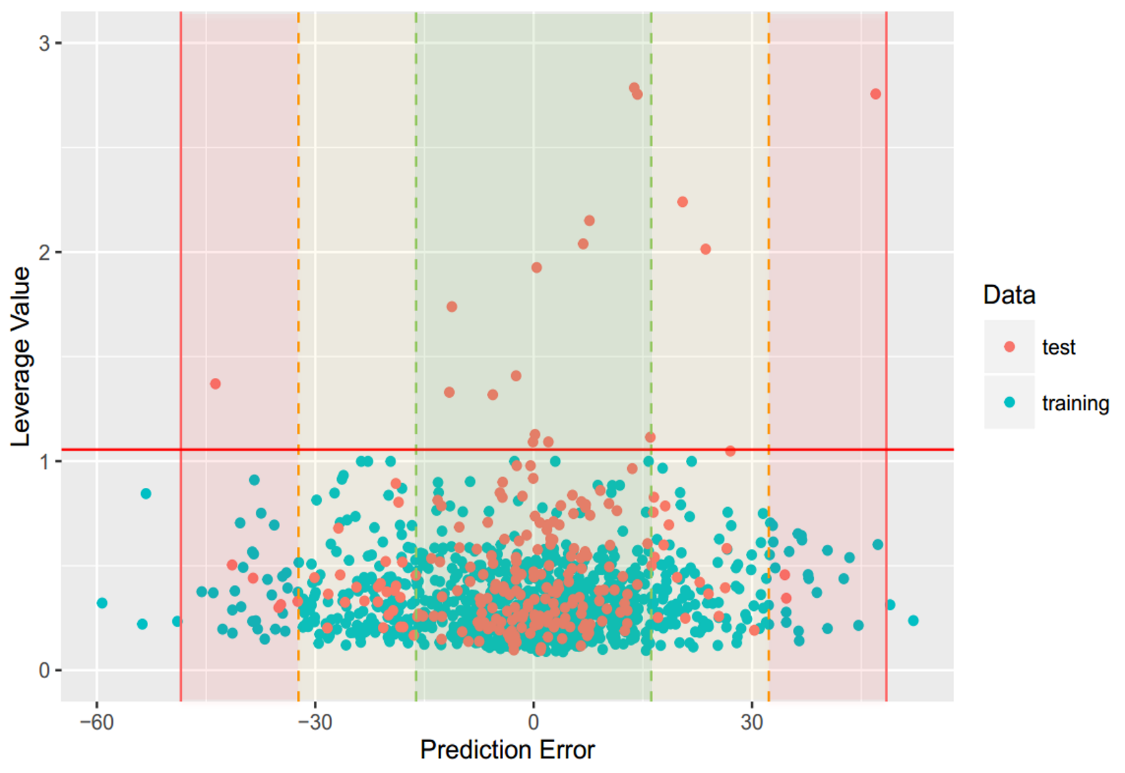
**

**Figure 8.** Williams plot of RF model

**VD:** For this property, four regression models for predicting VD were developed by RF, SVM, RP and PLS. The descriptors used in modeling process were listed in Table 15 and the statistic results for four models can be seen in Table 16. The plot of predicted VD versus experimental VD for the training set and the test set is shown in Figure 9. From the Table 16 and Figure 9, we can see that the regression model using RF was the best one (Q^2^=0.634, R_T_^2^=0.556). For this best model, the Williams plot was applied to define its application domain in Figure 10. As can be seen in this figure, the majority of compounds in the training and test set fall within the AD, indicating these compounds are most likely to be well predicted by the RF model.

**Table 15.** Selected descriptors in modeling process

| **Descriptors (45)** |
| --- |
| GMTIV, UI, MATSe1, MATSp1, Chiv4, MATSm2, S12, dchi3, IDE, PEOEVSA7, bcutp1, bcutm9, SIC1, MRVSA6, IC1, QNmax, CIC0, PEOEVSA6, MATSe4, VSAEstate8, Geto, EstateVSA3, MRVSA5, LogP2, Tnc, S7, SPP, QOmin, EstateVSA7, LogP, QNmin, MRVSA9, S19, MATSv2, nsulph, S17, S9, ndb, AWeight, QCss, EstateVSA9, Hy, S16, IC0, S30 |

**Table 16**. The statistic results of models built by RF, SVM, RP and PLS

| Method | Training size | Test size | mtry | $\mathbf{R}^{\mathbf{2}}$ | $\mathbf{Q}^{\mathbf{2}}$ | $\mathbf{R}_{\mathbf{T}}^{\mathbf{2}}$ | RMSE_F_ | RMSE_CV_ | RMSE_T_ |
| --- | --- | --- | --- | --- | --- | --- | --- | --- | --- |
| **RF** | **408** | **136** | **10** | **0.950** | **0.634** | **0.556** | **0.281** | **0.762** | **0.948** |
| SVM | 408 | 136 | - | 0.885 | 0.610 | 0.552 | 0.427 | 0.786 | 0.952 |
| RP | 408 | 136 | - | 0.768 | 0.268 | 0.366 | 0.606 | 1.08 | 1.130 |
| PLS | 408 | 136 | - | 0.567 | 0.501 | 0.419 | 0.829 | 0.89 | 1.080 |
| RF | 408 | 136 | 10 | 2-fold rate(cv/test) 0.819/0.801 | | | 3-fold rate(cv/test)  0.912/0.904 | | |


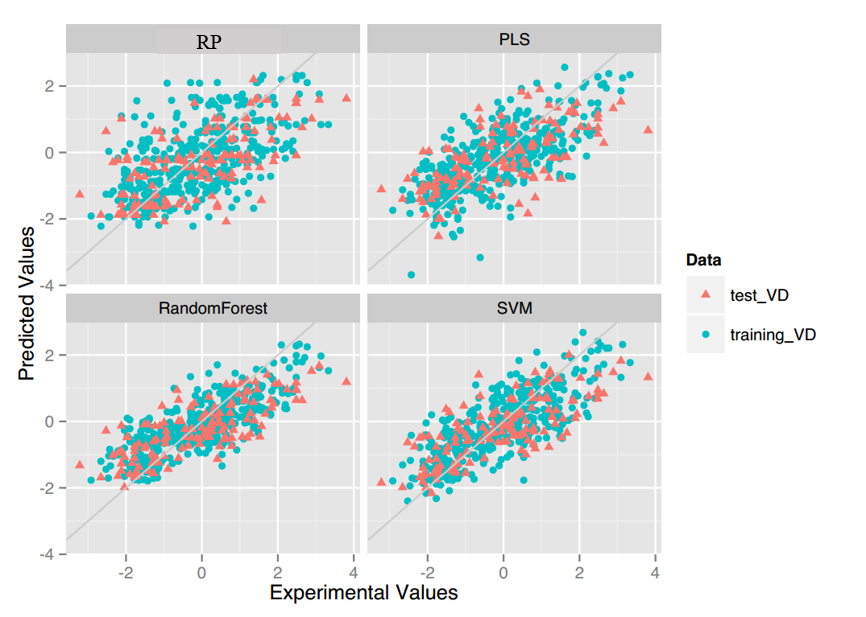


**Figure 9**. Plot of predicted values versus experimental values of models using four methods.


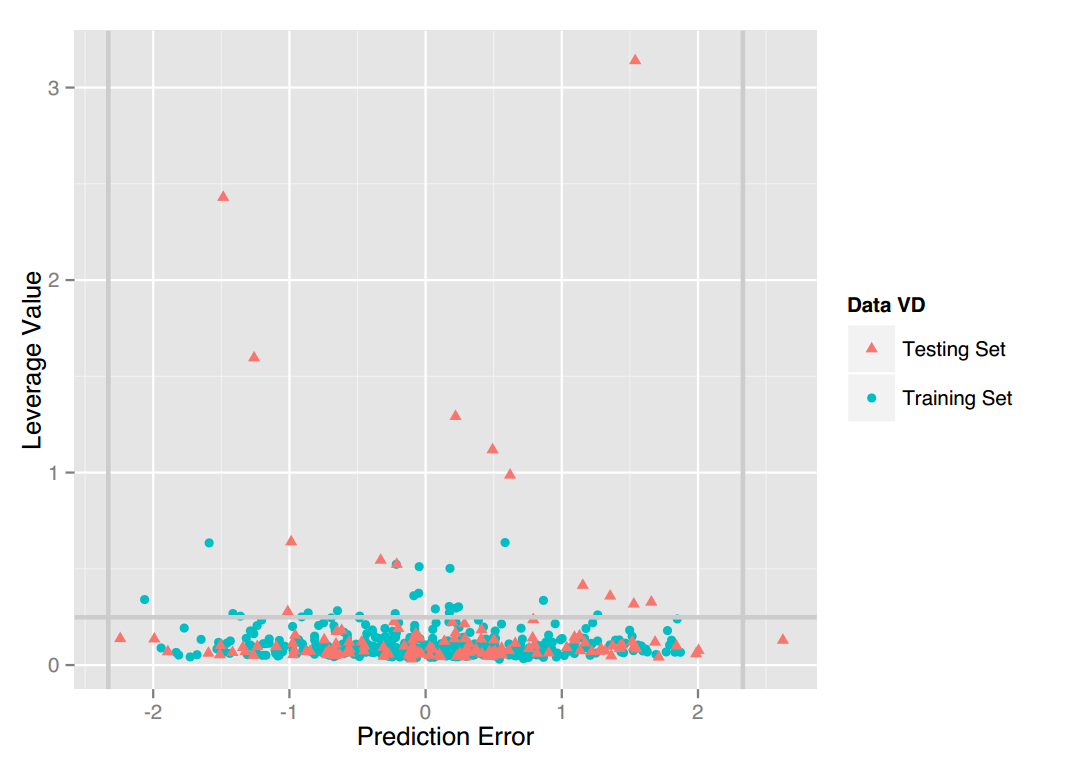


**Figure 10.** Williams plot of RF model.

Considering the barely satisfactory results of this property, the percentage of compounds predicted within different fold error (Fold) was applied to assess model performance. They are defined as follows: fold= 1+|Y_pred_-Y_true_|/Y_true_. A prediction method with an average-fold error <2 was considered successful. The statistic results based on RF and same descriptors were also listed in Table 16. From this table, we can see that 81.9% of training compounds and 80.1% of test compounds are within 2-fold error for VD prediction. Compared with similar study published in 2009(2-fold error: 67% for training set, 66% for test set), our model performs somewhat better and may be more practical in future application.[[62](#_ENREF_62)] Corresponding fold-rate relationship can be seen in Figure 11.

**
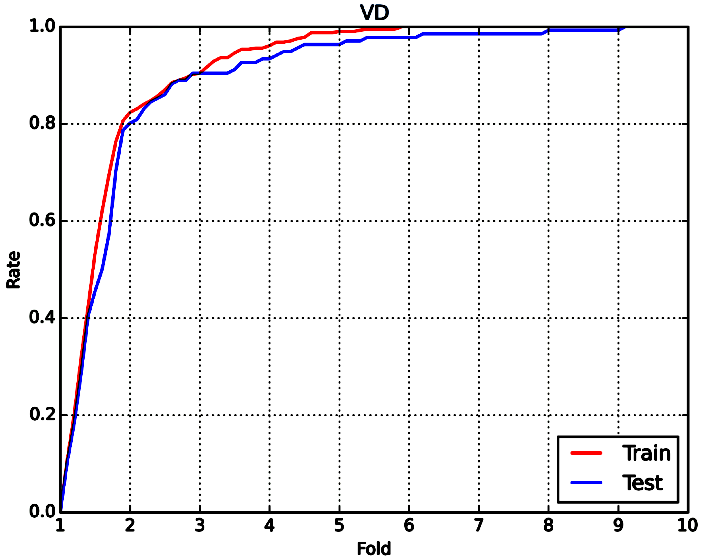
**

**Figure 11.** The fold-rate relationship of VD prediction

**BBB:** For this property, 20 classification models were developed by four methods (RF, SVM, NB, DT) and five fingerprints (FP2, MACCS, ECFP2, ECFP4, ECFP6). The statistic results for these classification models can be seen in Table 17. From the table 17, we can see that the classification model based on SVM and ECFP2 was the best one with ACC=0.926 for the training set and ACC=0.962 for the test set. Compared with the prediction model developed by Hu Li (ACC=83.7% for training set, ACC=85.4% for test set), our classification model has a better predictive ability in the perspective of statistics.[[63](#_ENREF_63)]

**Table 17**. The statistic results of different classification models

| Method | fingerprint | Five folds cross validation | | | | External validation dataset | | | |
| --- | --- | --- | --- | --- | --- | --- | --- | --- | --- |
|  |  | Sensitivity | Specificity | Accuracy | AUC | Sensitivity | Specificity | Accuracy | AUC |
| DT | FP2 | 0.879 | 0.773 | 0.853 | 0.830 | 0.893 | 0.878 | 0.890 | 0.888 |
|  | MACCS | 0.922 | 0.793 | 0.890 | 0.860 | 0.953 | 0.870 | 0.935 | 0.921 |
|  | ECFP2 | 0.929 | 0.773 | 0.891 | 0.855 | 0.935 | 0.886 | 0.924 | 0.914 |
|  | ECFP4 | 0.914 | 0.788 | 0.883 | 0.854 | 0.909 | 0.878 | 0.902 | 0.895 |
|  | ECFP6 | 0.915 | 0.764 | 0.878 | 0.842 | 0.947 | 0.854 | 0.926 | 0.902 |
| BNB | FP2 | 0.706 | 0.660 | 0.695 | 0.686 | 0.728 | 0.675 | 0.716 | 0.712 |
|  | MACCS | 0.877 | 0.663 | 0.824 | 0.851 | 0.881 | 0.691 | 0.839 | 0.867 |
|  | ECFP2 | 0.974 | 0.606 | 0.884 | 0.914 | 0.960 | 0.634 | 0.888 | 0.916 |
|  | ECFP4 | 0.964 | 0.640 | 0.885 | 0.924 | 0.967 | 0.699 | 0.908 | 0.932 |
|  | ECFP6 | 0.968 | 0.670 | 0.895 | 0.910 | 0.970 | 0.675 | 0.904 | 0.920 |
| SVM | FP2^a^ | 0.976 | 0.754 | 0.921 | 0.940 | 0.986 | 0.724 | 0.928 | 0.950 |
|  | MACCS^b^ | 0.953 | 0.823 | 0.921 | 0.949 | 0.986 | 0.902 | 0.967 | 0.973 |
|  | **ECFP2^c^** | **0.962** | **0.813** | **0.926** | **0.948** | **0.993** | **0.854** | **0.962** | **0.975** |
|  | ECFP4^d^ | 0.963 | 0.820 | 0.928 | 0.950 | 0.993 | 0.846 | 0.960 | 0.972 |
|  | ECFP6^e^ | 0.963 | 0.808 | 0.925 | 0.947 | 0.988 | 0.854 | 0.958 | 0.972 |
| RF | FP2^f^ | 0.978 | 0.719 | 0.914 | 0.934 | 0.986 | 0.813 | 0.948 | 0.967 |
|  | MACCS^g^ | 0.978 | 0.788 | 0.931 | 0.959 | 1.000 | 0.870 | 0.971 | 0.979 |
|  | ECFP2^h^ | 0.981 | 0.741 | 0.922 | 0.960 | 1.000 | 0.813 | 0.958 | 0.975 |
|  | ECFP4^i^ | 0.980 | 0.756 | 0.925 | 0.957 | 1.000 | 0.829 | 0.962 | 0.974 |
|  | ECFP6^j^ | 0.983 | 0.709 | 0.916 | 0.952 | 1.000 | 0.772 | 0.949 | 0.972 |

a Coarse grid-search best: C = 2^1^, gamma = 2^-9^, finer grid-search best: C = 2^2^, gamma= 2^-9^

b Coarse grid-search best: C = 2^5^, gamma =2^-7^, finer grid-search best: C = 2^3.75^, gamma=2^-6^

c Coarse grid-search best: C = 2^3^, gamma = 2^-5^, finer grid-search best: C=2^2^, gamma=2^-5^

d Coarse grid-search best: C = 2^5^, gamma = 2^-9^, finer grid-search best: C = 2^4^, gamma = 2^-8.5^

e Coarse grid-search best: C = 2^11^, gamma = 2^-7^, finer grid-search best: C = 2^12.5^ , gamma = 2^-7^

f mtry = 10

g mtry = 10

h mtry = 10

i mtry = 20

j mtry = 10

- 1. ****Metabolism****

**CYP 1A2-Inhibitor:** For this property, 20 classification models were developed by RF, SVM, NB, DT and FP2, MACCS, ECFP2, ECFP4, ECFP6. The statistic results for these classification models can be seen in Table 18. From the table 18, we can see that the classification model based on SVM and ECFP4 was the best one with ACC=0.849 for the training set and ACC=0.867 for the test set.

**Table 18**. The statistic results of different classification models

| Method | fingerprint | Five folds cross validation | | | | External validation dataset | | | |
| --- | --- | --- | --- | --- | --- | --- | --- | --- | --- |
|  |  | Sensitivity | Specificity | Accuracy | AUC | Sensitivity | Specificity | Accuracy | AUC |
| DT | FP2 | 0.700 | 0.721 | 0.711 | 0.710 | 0.676 | 0.725 | 0.702 | 0.700 |
|  | MACCS | 0.741 | 0.782 | 0.763 | 0.763 | 0.746 | 0.784 | 0.766 | 0.766 |
|  | ECFP2 | 0.756 | 0.782 | 0.770 | 0.770 | 0.797 | 0.794 | 0.795 | 0.795 |
|  | ECFP4 | 0.745 | 0.777 | 0.762 | 0.761 | 0.748 | 0.797 | 0.774 | 0.772 |
|  | ECFP6 | 0.727 | 0.751 | 0.740 | 0.739 | 0.732 | 0.776 | 0.755 | 0.754 |
| BNB | FP2 | 0.626 | 0.701 | 0.665 | 0.684 | 0.638 | 0.702 | 0.672 | 0.692 |
|  | MACCS | 0.752 | 0.755 | 0.754 | 0.828 | 0.790 | 0.741 | 0.764 | 0.842 |
|  | ECFP2 | 0.807 | 0.755 | 0.780 | 0.861 | 0.819 | 0.764 | 0.790 | 0.875 |
|  | ECFP4 | 0.758 | 0.793 | 0.777 | 0.860 | 0.784 | 0.808 | 0.797 | 0.877 |
|  | ECFP6 | 0.735 | 0.800 | 0.770 | 0.852 | 0.749 | 0.823 | 0.788 | 0.872 |
| SVM | FP2^a^ | 0.808 | 0.844 | 0.827 | 0.905 | 0.845 | 0.847 | 0.846 | 0.925 |
|  | MACCS^b^ | 0.816 | 0.849 | 0.834 | 0.911 | 0.836 | 0.858 | 0.848 | 0.922 |
|  | ECFP2^c^ | 0.836 | 0.859 | 0.848 | 0.924 | 0.863 | 0.871 | 0.867 | 0.936 |
|  | **ECFP4^d^** | **0.833** | **0.864** | **0.849** | **0.928** | **0.853** | **0.880** | **0.867** | **0.939** |
|  | ECFP6^e^ | 0.825 | 0.857 | 0.842 | 0.923 | 0.838 | 0.874 | 0.857 | 0.933 |
| RF | FP2^f^ | 0.787 | 0.835 | 0.812 | 0.896 | 0.822 | 0.838 | 0.831 | 0.913 |
|  | MACCS^g^ | 0.800 | 0.851 | 0.827 | 0.908 | 0.815 | 0.857 | 0.837 | 0.919 |
|  | ECFP2^h^ | 0.825 | 0.839 | 0.832 | 0.913 | 0.838 | 0.854 | 0.847 | 0.928 |
|  | ECFP4^i^ | 0.818 | 0.849 | 0.834 | 0.914 | 0.838 | 0.863 | 0.852 | 0.928 |
|  | ECFP6^j^ | 0.800 | 0.850 | 0.826 | 0.912 | 0.829 | 0.870 | 0.851 | 0.924 |

a Coarse grid-search best: C = 2^1^, gamma = 2^-9^, finer grid-search best: C = 2^1^, gamma=2^-8.5^

b Coarse grid-search best: C = 2^1^, gamma =2^-3^, finer grid-search best: C = 2^0.5^, gamma=2^-3.5^

c Coarse grid-search best: C = 2^1^, gamma = 2^-3^, finer grid-search best: C=2^1^, gamma=2^-3.5^

d Coarse grid-search best: C = 2^1^, gamma = 2^-5^, finer grid-search best: C = 2^1.5^, gamma=2^-4. 5^

e Coarse grid-search best: C = 2^1^, gamma = 2^-5^, finer grid-search best: C = 2^1^ , gamma = 2^-5^

f mtry = 270

g mtry = 40

h mtry = 30

i mtry = 20

j mtry = 60

**CYP 2C19-Inhibitor:** For this property, 20 classification models were developed by RF, SVM, NB, DT and FP2, MACCS, ECFP2, ECFP4, ECFP6. The statistic results for these classification models can be seen in Table 19. From the table 19, we can see that the classification model based on SVM and ECFP2 was the best one with ACC=0.822 for the training set and ACC=0.819 for the test set.

**Table 19**. The statistic results of different classification models

| Method | fingerprint | Five folds cross validation | | | | External validation dataset | | | |
| --- | --- | --- | --- | --- | --- | --- | --- | --- | --- |
|  |  | Sensitivity | Specificity | Accuracy | AUC | Sensitivity | Specificity | Accuracy | AUC |
| DT | FP2 | 0.641 | 0.706 | 0.676 | 0.673 | 0.649 | 0.722 | 0.689 | 0.685 |
|  | MACCS | 0.682 | 0.743 | 0.715 | 0.715 | 0.710 | 0.759 | 0.736 | 0.736 |
|  | ECFP2 | 0.714 | 0.756 | 0.737 | 0.736 | 0.694 | 0.763 | 0.731 | 0.729 |
|  | ECFP4 | 0.692 | 0.748 | 0.722 | 0.720 | 0.703 | 0.743 | 0.725 | 0.723 |
|  | ECFP6 | 0.664 | 0.730 | 0.700 | 0.697 | 0.689 | 0.725 | 0.708 | 0.707 |
| BNB | FP2 | 0.713 | 0.526 | 0.612 | 0.632 | 0.708 | 0.551 | 0.624 | 0.639 |
|  | MACCS | 0.695 | 0.678 | 0.686 | 0.757 | 0.677 | 0.692 | 0.685 | 0.762 |
|  | ECFP2 | 0.798 | 0.720 | 0.756 | 0.827 | 0.791 | 0.725 | 0.755 | 0.826 |
|  | ECFP4 | 0.804 | 0.703 | 0.750 | 0.829 | 0.807 | 0.717 | 0.759 | 0.831 |
|  | ECFP6 | 0.819 | 0.697 | 0.753 | 0.828 | 0.807 | 0.692 | 0.745 | 0.828 |
| SVM | FP2^a^ | 0.788 | 0.786 | 0.787 | 0.863 | 0.787 | 0.792 | 0.790 | 0.867 |
|  | MACCS^b^ | 0.803 | 0.804 | 0.803 | 0.873 | 0.797 | 0.817 | 0.807 | 0.881 |
|  | **ECFP2^c^** | **0.826** | **0.819** | **0.822** | **0.893** | **0.812** | **0.825** | **0.819** | **0.899** |
|  | ECFP4^d^ | 0.823 | 0.823 | 0.823 | 0.896 | 0.815 | 0.820 | 0.818 | 0.896 |
|  | ECFP6^e^ | 0.833 | 0.807 | 0.819 | 0.892 | 0.825 | 0.809 | 0.816 | 0.893 |
| RF | FP2^f^ | 0.805 | 0.742 | 0.771 | 0.850 | 0.807 | 0.758 | 0.781 | 0.860 |
|  | MACCS^g^ | 0.801 | 0.789 | 0.795 | 0.865 | 0.802 | 0.798 | 0.800 | 0.876 |
|  | ECFP2^h^ | 0.830 | 0.793 | 0.810 | 0.884 | 0.821 | 0.809 | 0.815 | 0.889 |
|  | ECFP4^i^ | 0.820 | 0.797 | 0.807 | 0.885 | 0.823 | 0.803 | 0.812 | 0.888 |
|  | ECFP6^j^ | 0.801 | 0.803 | 0.802 | 0.881 | 0.797 | 0.820 | 0.809 | 0.886 |

a Coarse grid-search best: C = 2^1^, gamma = 2^-9^, finer grid-search best: C = 2^0.5^, gamma=2^-8.5^

b Coarse grid-search best: C = 2^0^, gamma =2^-3^, finer grid-search best: C = 2^0^, gamma=2^-3.5^

c Coarse grid-search best: C = 2^1^, gamma = 2^-5^, finer grid-search best: C=2^1^, gamma=2^-4.5^

d Coarse grid-search best: C = 2^1^, gamma = 2^-5^, finer grid-search best: C = 2^1^, gamma=2^-5^

e Coarse grid-search best: C = 2^1^, gamma = 2^-5^, finer grid-search best: C = 2^0.5^, gamma = 2^-5^

f mtry = 280

g mtry = 20

h mtry = 40

i mtry = 20

j mtry = 10

**CYP 2C9-Inhibitor:** For this property, 20 classification models were developed by RF, SVM, NB, DT and FP2, MACCS, ECFP2, ECFP4, ECFP6. The statistic results for these classification models can be seen in Table 20. From the table 20, we can see that the classification model based on SVM and ECFP4 was the best one with ACC=0.837 for the training set and ACC=0.830 for the test set.

**Table 20**. The statistic results of different classification models

| Method | fingerprint | Five folds cross validation | | | | External validation dataset | | | |
| --- | --- | --- | --- | --- | --- | --- | --- | --- | --- |
|  |  | Sensitivity | Specificity | Accuracy | AUC | Sensitivity | Specificity | Accuracy | AUC |
| DT | FP2 | 0.575 | 0.770 | 0.704 | 0.672 | 0.577 | 0.773 | 0.706 | 0.675 |
|  | MACCS | 0.611 | 0.799 | 0.736 | 0.707 | 0.597 | 0.799 | 0.730 | 0.703 |
|  | ECFP2 | 0.601 | 0.806 | 0.737 | 0.704 | 0.597 | 0.802 | 0.732 | 0.700 |
|  | ECFP4 | 0.605 | 0.793 | 0.730 | 0.699 | 0.620 | 0.779 | 0.725 | 0.699 |
|  | ECFP6 | 0.579 | 0.789 | 0.718 | 0.684 | 0.579 | 0.781 | 0.713 | 0.680 |
| BNB | FP2 | 0.720 | 0.608 | 0.646 | 0.671 | 0.699 | 0.602 | 0.635 | 0.663 |
|  | MACCS | 0.649 | 0.719 | 0.695 | 0.758 | 0.634 | 0.722 | 0.692 | 0.755 |
|  | ECFP2 | 0.744 | 0.778 | 0.767 | 0.834 | 0.752 | 0.778 | 0.769 | 0.834 |
|  | ECFP4 | 0.747 | 0.778 | 0.767 | 0.841 | 0.747 | 0.770 | 0.762 | 0.834 |
|  | ECFP6 | 0.747 | 0.792 | 0.777 | 0.844 | 0.727 | 0.777 | 0.760 | 0.832 |
| SVM | FP2^a^ | 0.698 | 0.871 | 0.813 | 0.880 | 0.703 | 0.856 | 0.804 | 0.868 |
|  | MACCS^b^ | 0.677 | 0.873 | 0.807 | 0.871 | 0.684 | 0.853 | 0.796 | 0.867 |
|  | ECFP2^c^ | 0.707 | 0.891 | 0.829 | 0.895 | 0.712 | 0.878 | 0.821 | 0.890 |
|  | **ECFP4^d^** | **0.719** | **0.898** | **0.837** | **0.900** | **0.730** | **0.882** | **0.830** | **0.894** |
|  | ECFP6^e^ | 0.717 | 0.892 | 0.833 | 0.898 | 0.718 | 0.884 | 0.827 | 0.889 |
| RF | FP2^f^ | 0.627 | 0.890 | 0.801 | 0.869 | 0.638 | 0.883 | 0.800 | 0.864 |
|  | MACCS^g^ | 0.655 | 0.870 | 0.797 | 0.866 | 0.666 | 0.860 | 0.794 | 0.861 |
|  | ECFP2^h^ | 0.579 | 0.921 | 0.806 | 0.883 | 0.603 | 0.907 | 0.804 | 0.876 |
|  | ECFP4^i^ | 0.497 | 0.952 | 0.798 | 0.893 | 0.503 | 0.942 | 0.793 | 0.884 |
|  | ECFP6^j^ | 0.597 | 0.922 | 0.813 | 0.888 | 0.593 | 0.914 | 0.805 | 0.875 |

a Coarse grid-search best: C = 2^3^, gamma = 2^-9^, finer grid-search best: C = 2^2.5^, gamma=2^-8.5^

b Coarse grid-search best: C = 2^1^, gamma =2^-5^, finer grid-search best: C = 2^1^, gamma=2^-4.5^

c Coarse grid-search best: C = 2^1^, gamma = 2^-5^, finer grid-search best: C=2^1^, gamma=2^-4.5^

d Coarse grid-search best: C = 2^1^, gamma = 2^-5^, finer grid-search best: C = 2^1^, gamma=2^-4.5^

e Coarse grid-search best: C = 2^7^, gamma = 2^-5^, finer grid-search best: C = 2^7^ , gamma = 2^-5^

f mtry = 250

g mtry = 40

h mtry = 30

i mtry = 10

j mtry = 60

**CYP 2D6-Inhibitor:** For this property, 20 classification models were developed by RF, SVM, NB, DT and FP2, MACCS, ECFP2, ECFP4, ECFP6. The statistic results for these classification models can be seen in Table 21. From the table 21, we can see that the classification models based on SVM, NB, DT are unbalanced. Therefore, some balanced models are built and the model based on RF and ECFP4 was the best one with ACC=0.793 for the training set and ACC=0.795 for the test set.

**Table 21**. The statistic results of different classification models

| Method | fingerprint | Five folds cross validation | | | | External validation dataset | | | |
| --- | --- | --- | --- | --- | --- | --- | --- | --- | --- |
|  |  | Sensitivity | Specificity | Accuracy | AUC | Sensitivity | Specificity | Accuracy | AUC |
| DT | FP2 | 0.466 | 0.843 | 0.773 | 0.655 | 0.427 | 0.847 | 0.773 | 0.637 |
|  | MACCS | 0.525 | 0.866 | 0.802 | 0.703 | 0.452 | 0.877 | 0.802 | 0.667 |
|  | ECFP2 | 0.501 | 0.901 | 0.826 | 0.702 | 0.491 | 0.901 | 0.829 | 0.697 |
|  | ECFP4 | 0.498 | 0.890 | 0.817 | 0.694 | 0.471 | 0.892 | 0.818 | 0.681 |
|  | ECFP6 | 0.481 | 0.893 | 0.816 | 0.687 | 0.484 | 0.900 | 0.827 | 0.692 |
| BNB | FP2 | 0.636 | 0.572 | 0.584 | 0.616 | 0.588 | 0.578 | 0.580 | 0.592 |
|  | MACCS | 0.589 | 0.782 | 0.746 | 0.750 | 0.594 | 0.808 | 0.771 | 0.754 |
|  | ECFP2 | 0.592 | 0.874 | 0.822 | 0.815 | 0.560 | 0.883 | 0.826 | 0.803 |
|  | ECFP4 | 0.589 | 0.868 | 0.816 | 0.813 | 0.554 | 0.869 | 0.814 | 0.802 |
|  | ECFP6 | 0.552 | 0.882 | 0.820 | 0.808 | 0.529 | 0.890 | 0.826 | 0.796 |
| SVM | FP2^a^ | 0.432 | 0.966 | 0.866 | 0.848 | 0.438 | 0.970 | 0.876 | 0.834 |
|  | MACCS^b^ | 0.386 | 0.974 | 0.864 | 0.849 | 0.374 | 0.981 | 0.874 | 0.839 |
|  | ECFP2^c^ | 0.483 | 0.969 | 0.878 | 0.865 | 0.444 | 0.972 | 0.880 | 0.871 |
|  | ECFP4^d^ | 0.464 | 0.973 | 0.878 | 0.874 | 0.431 | 0.978 | 0.882 | 0.873 |
|  | ECFP6^e^ | 0.429 | 0.975 | 0.873 | 0.873 | 0.404 | 0.980 | 0.879 | 0.869 |
| RF | FP2^f^ | 0.313 | 0.981 | 0.856 | 0.829 | 0.306 | 0.985 | 0.866 | 0.817 |
|  | MACCS^g^ | 0.437 | 0.964 | 0.866 | 0.855 | 0.389 | 0.972 | 0.870 | 0.849 |
|  | ECFP2^h^ | 0.370 | 0.978 | 0.864 | 0.869 | 0.351 | 0.982 | 0.871 | 0.862 |
|  | ECFP4^i^ | 0.311 | 0.986 | 0.860 | 0.872 | 0.287 | 0.989 | 0.865 | 0.867 |
|  | ECFP6^j^ | 0.305 | 0.986 | 0.859 | 0.866 | 0.290 | 0.987 | 0.865 | 0.864 |
|  | **ECFP4** | **0.770** | **0.811** | **0.793** | **0.868** | **0.771** | **0.812** | **0.795** | **0.882** |

a Coarse grid-search best: C = 2^1^, gamma = 2^-9^, finer grid-search best: C = 2^1.5^, gamma=2^-8.5^

b Coarse grid-search best: C = 2^0^, gamma =2^-3^, finer grid-search best: C = 2^0.5^, gamma=2^-3.5^

c Coarse grid-search best: C = 2^1^, gamma = 2^-5^, finer grid-search best: C=2^1^, gamma=2^-4.5^

d Coarse grid-search best: C = 2^1^, gamma = 2^-5^, finer grid-search best: C = 2^1^, gamma=2^-4.5^

e Coarse grid-search best: C = 2^1^, gamma = 2^-5^, finer grid-search best: C = 2^1^ , gamma = 2^-5^

f mtry = 180

g mtry = 40

h mtry = 40

i mtry = 10

j mtry = 20

**CYP 3A4-Inhibitor:** For this property, 20 classification models were developed by RF, SVM, NB, DT and FP2, MACCS, ECFP2, ECFP4, ECFP6. The statistic results for these classification models can be seen in Table 22. From the table 22, we can see that the classification model based on SVM and ECFP4 was the best one with ACC=0.817 for the training set and ACC=0.829 for the test set.

For the 5 CYP-inhibitor prediction models, their whole accuracy value was in the range of 0.793-0.849 for training set and 0.795-0.867 for test set. Compared with the statistic result of latest web tool, SwissADME, (the whole accuracy value was in the range of 0.77-0.83 for training set and 0.78-0.84 for test set), our classification models were comparable and even better.[[64](#_ENREF_64)]

**Table 22**. The statistic results of different classification models

| Method | fingerprint | Five folds cross validation | | | | External validation dataset | | | |
| --- | --- | --- | --- | --- | --- | --- | --- | --- | --- |
|  |  | Sensitivity | Specificity | Accuracy | AUC | Sensitivity | Specificity | Accuracy | AUC |
| DT | FP2 | 0.632 | 0.715 | 0.680 | 0.674 | 0.653 | 0.721 | 0.692 | 0.687 |
|  | MACCS | 0.649 | 0.737 | 0.700 | 0.697 | 0.670 | 0.736 | 0.707 | 0.708 |
|  | ECFP2 | 0.673 | 0.771 | 0.729 | 0.722 | 0.716 | 0.775 | 0.750 | 0.746 |
|  | ECFP4 | 0.664 | 0.756 | 0.717 | 0.710 | 0.690 | 0.750 | 0.724 | 0.720 |
|  | ECFP6 | 0.648 | 0.751 | 0.708 | 0.700 | 0.656 | 0.769 | 0.720 | 0.713 |
| BNB | FP2 | 0.753 | 0.568 | 0.646 | 0.669 | 0.756 | 0.562 | 0.646 | 0.672 |
|  | MACCS | 0.739 | 0.621 | 0.671 | 0.733 | 0.742 | 0.604 | 0.664 | 0.731 |
|  | ECFP2 | 0.763 | 0.706 | 0.730 | 0.819 | 0.773 | 0.710 | 0.737 | 0.820 |
|  | ECFP4 | 0.690 | 0.800 | 0.753 | 0.831 | 0.708 | 0.797 | 0.758 | 0.838 |
|  | ECFP6 | 0.684 | 0.821 | 0.763 | 0.838 | 0.691 | 0.816 | 0.763 | 0.844 |
| SVM | FP2^a^ | 0.712 | 0.837 | 0.784 | 0.865 | 0.722 | 0.853 | 0.797 | 0.877 |
|  | MACCS^b^ | 0.734 | 0.814 | 0.780 | 0.861 | 0.736 | 0.812 | 0.780 | 0.861 |
|  | ECFP2^c^ | 0.751 | 0.846 | 0.806 | 0.893 | 0.787 | 0.861 | 0.829 | 0.906 |
|  | **ECFP4^d^** | **0.759** | **0.858** | **0.817** | **0.901** | **0.788** | **0.860** | **0.829** | **0.909** |
|  | ECFP6^e^ | 0.765 | 0.850 | 0.814 | 0.896 | 0.788 | 0.857 | 0.827 | 0.906 |
| RF | FP2^f^ | 0.675 | 0.843 | 0.772 | 0.852 | 0.695 | 0.855 | 0.786 | 0.865 |
|  | MACCS^g^ | 0.712 | 0.824 | 0.777 | 0.854 | 0.715 | 0.819 | 0.774 | 0.862 |
|  | ECFP2^h^ | 0.662 | 0.873 | 0.784 | 0.876 | 0.714 | 0.877 | 0.807 | 0.891 |
|  | ECFP4^i^ | 0.586 | 0.921 | 0.779 | 0.882 | 0.631 | 0.919 | 0.795 | 0.896 |
|  | ECFP6^j^ | 0.552 | 0.932 | 0.771 | 0.881 | 0.597 | 0.930 | 0.787 | 0.897 |

a Coarse grid-search best: C = 2^1^, gamma = 2^-9^, finer grid-search best: C = 2^1^, gamma=2^-8.5^

b Coarse grid-search best: C = 2^0^, gamma =2^-5^, finer grid-search best: C = 2^0.5^, gamma=2^-4.5^

c Coarse grid-search best: C = 2^1^, gamma = 2^-5^, finer grid-search best: C=2^1^, gamma=2^-4.5^

d Coarse grid-search best: C = 2^1^, gamma = 2^-5^, finer grid-search best: C = 2^1^, gamma=2^-5^

e Coarse grid-search best: C = 2^3^, gamma = 2^-5^, finer grid-search best: C = 2^2.5^, gamma = 2^-5^

f mtry= 300

g Mtry = 30

h mtry = 60

i mtry = 20

j mtry = 10

**CYP 2C9-Substrate:** For this property, 20 classification models were developed by RF, SVM, NB, DT and FP2, MACCS, ECFP2, ECFP4, ECFP6. The statistic results for these classification models can be seen in Table 23. From the table 23, we can see that this dataset was also unbalanced. Thus, the balanced classification model based on RF and ECFP4 was the best one with ACC=0.728 for the training set and ACC=0.734 for the test set.

**Table 23**. The statistic results of different classification models

| Method | fingerprint | Five folds cross validation | | | | External validation dataset | | | |
| --- | --- | --- | --- | --- | --- | --- | --- | --- | --- |
|  |  | Sensitivity | Specificity | Accuracy | AUC | Sensitivity | Specificity | Accuracy | AUC |
| DT | FP2 | 0.697 | 0.510 | 0.634 | 0.603 | 0.729 | 0.427 | 0.611 | 0.576 |
|  | MACCS | 0.736 | 0.582 | 0.684 | 0.661 | 0.644 | 0.547 | 0.606 | 0.603 |
|  | ECFP2 | 0.713 | 0.485 | 0.636 | 0.599 | 0.737 | 0.507 | 0.648 | 0.620 |
|  | ECFP4 | 0.731 | 0.485 | 0.648 | 0.607 | 0.737 | 0.547 | 0.663 | 0.640 |
|  | ECFP6 | 0.726 | 0.510 | 0.653 | 0.617 | 0.720 | 0.480 | 0.627 | 0.598 |
| BNB | FP2 | 0.520 | 0.617 | 0.553 | 0.577 | 0.534 | 0.547 | 0.539 | 0.579 |
|  | MACCS | 0.721 | 0.531 | 0.656 | 0.686 | 0.695 | 0.480 | 0.611 | 0.639 |
|  | ECFP2 | 0.911 | 0.301 | 0.705 | 0.698 | 0.856 | 0.453 | 0.699 | 0.772 |
|  | ECFP4 | 0.731 | 0.617 | 0.693 | 0.737 | 0.703 | 0.613 | 0.668 | 0.770 |
|  | ECFP6 | 0.721 | 0.577 | 0.672 | 0.734 | 0.686 | 0.573 | 0.642 | 0.727 |
| SVM | FP2^a^ | 0.877 | 0.439 | 0.729 | 0.757 | 0.847 | 0.413 | 0.679 | 0.721 |
|  | MACCS^b^ | 0.888 | 0.418 | 0.729 | 0.753 | 0.907 | 0.240 | 0.648 | 0.657 |
|  | ECFP2^c^ | 0.869 | 0.485 | 0.739 | 0.758 | 0.839 | 0.480 | 0.699 | 0.723 |
|  | ECFP4^d^ | 0.919 | 0.423 | 0.751 | 0.774 | 0.915 | 0.427 | 0.725 | 0.746 |
|  | ECFP6^e^ | 0.903 | 0.454 | 0.751 | 0.770 | 0.907 | 0.480 | 0.741 | 0.744 |
| RF | FP2^f^ | 0.919 | 0.372 | 0.734 | 0.755 | 0.873 | 0.333 | 0.663 | 0.734 |
|  | MACCS^g^ | 0.833 | 0.490 | 0.717 | 0.743 | 0.831 | 0.453 | 0.684 | 0.708 |
|  | ECFP2^h^ | 0.893 | 0.408 | 0.729 | 0.747 | 0.890 | 0.467 | 0.725 | 0.772 |
|  | ECFP4^i^ | 0.930 | 0.352 | 0.734 | 0.752 | 0.907 | 0.400 | 0.710 | 0.768 |
|  | ECFP6^j^ | 0.935 | 0.337 | 0.732 | 0.742 | 0.907 | 0.387 | 0.705 | 0.731 |
|  | **ECFP4** | **0.746** | **0.709** | **0.728** | **0.819** | **0.746** | **0.709** | **0.734** | **0.824** |

a Coarse grid-search best: C = 2^3^, gamma = 2^-9^, finer grid-search best: C = 2^3^, gamma= 2^-8.75^

b Coarse grid-search best: C = 2^9^, gamma =2^-15^, finer grid-search best: C = 2^9^, gamma=2^-15.25^

c Coarse grid-search best: C = 2^3^, gamma = 2^-5^, finer grid-search best: C=2^1.75^, gamma=2^-4.25^

d Coarse grid-search best: C = 2^1^, gamma = 2^-5^, finer grid-search best: C = 2^1^, gamma = 2^-4.75^

e Coarse grid-search best: C = 2^1^, gamma = 2^-5^, finer grid-search best: C = 2^2^ , gamma = 2^-6^

f mtry = 300

g mtry = 150

h mtry = 40

i mtry = 10

j mtry = 10

**CYP 2D6-Substrate:** For this property, 20 classification models were developed by RF, SVM, NB, DT and FP2, MACCS, ECFP2, ECFP4, ECFP6. The statistic results for these classification models can be seen in Table 24. From the table 24, we can see that this dataset was also unbalanced and thus the classification model based on RF and ECFP4 was the best one with ACC=0.748 for the training set and ACC=0.760 for the test set.

**Table 24**. The statistic results of different classification models

| Method | fingerprint | Five folds cross validation | | | | External validation dataset | | | |
| --- | --- | --- | --- | --- | --- | --- | --- | --- | --- |
|  |  | Sensitivity | Specificity | Accuracy | AUC | Sensitivity | Specificity | Accuracy | AUC |
| DT | FP2 | 0.647 | 0.563 | 0.610 | 0.605 | 0.612 | 0.600 | 0.607 | 0.608 |
|  | MACCS | 0.599 | 0.612 | 0.605 | 0.608 | 0.653 | 0.650 | 0.652 | 0.654 |
|  | ECFP2 | 0.712 | 0.612 | 0.668 | 0.663 | 0.661 | 0.675 | 0.667 | 0.668 |
|  | ECFP4 | 0.665 | 0.548 | 0.613 | 0.605 | 0.727 | 0.688 | 0.711 | 0.707 |
|  | ECFP6 | 0.665 | 0.574 | 0.625 | 0.619 | 0.645 | 0.650 | 0.647 | 0.647 |
| BNB | FP2 | 0.558 | 0.582 | 0.568 | 0.580 | 0.620 | 0.588 | 0.607 | 0.629 |
|  | MACCS | 0.656 | 0.707 | 0.678 | 0.724 | 0.620 | 0.838 | 0.706 | 0.818 |
|  | ECFP2 | 0.709 | 0.692 | 0.702 | 0.757 | 0.711 | 0.750 | 0.726 | 0.810 |
|  | ECFP4 | 0.659 | 0.719 | 0.685 | 0.760 | 0.669 | 0.738 | 0.697 | 0.804 |
|  | ECFP6 | 0.659 | 0.700 | 0.677 | 0.744 | 0.752 | 0.775 | 0.761 | 0.840 |
| SVM | FP2^a^ | 0.748 | 0.627 | 0.695 | 0.758 | 0.810 | 0.650 | 0.746 | 0.806 |
|  | MACCS^b^ | 0.837 | 0.574 | 0.722 | 0.782 | 0.851 | 0.750 | 0.811 | 0.854 |
|  | ECFP2^c^ | 0.825 | 0.620 | 0.735 | 0.797 | 0.868 | 0.713 | 0.806 | 0.848 |
|  | ECFP4^d^ | 0.846 | 0.582 | 0.730 | 0.802 | 0.884 | 0.700 | 0.811 | 0.847 |
|  | ECFP6^e^ | 0.822 | 0.620 | 0.733 | 0.796 | 0.868 | 0.663 | 0.786 | 0.842 |
| RF | FP2^f^ | 0.760 | 0.548 | 0.667 | 0.727 | 0.835 | 0.675 | 0.771 | 0.826 |
|  | MACCS^g^ | 0.751 | 0.658 | 0.710 | 0.778 | 0.843 | 0.750 | 0.806 | 0.860 |
|  | ECFP2^h^ | 0.763 | 0.646 | 0.712 | 0.784 | 0.835 | 0.725 | 0.791 | 0.847 |
|  | ECFP4^i^ | 0.792 | 0.608 | 0.712 | 0.773 | 0.818 | 0.738 | 0.786 | 0.817 |
|  | ECFP6^j^ | 0.780 | 0.616 | 0.708 | 0.765 | 0.826 | 0.738 | 0.791 | 0.818 |
|  | **ECFP4** | **0.765** | **0.73** | **0.748** | **0.823** | **0.792** | **0.73** | **0.76** | **0.833** |

a Coarse grid-search best: C = 2^5^, gamma = 2^-15^, finer grid-search best: C = 2^4.75^, gamma= 2^-14.25^

b Coarse grid-search best: C = 2^3^, gamma =2^-3^, finer grid-search best: C = 2^2^, gamma=2^-2.75^

c Coarse grid-search best: C = 2^0^, gamma = 2^-5^, finer grid-search best: C=2^0.5^, gamma=2^-3.5^

d Coarse grid-search best: C = 2^0^, gamma = 2^-5^, finer grid-search best: C = 2^0^, gamma = 2^-5^

e Coarse grid-search best: C = 2^1^, gamma = 2^-5^, finer grid-search best: C = 2^0.75^ , gamma = 2^-6.75^

f mtry = 1200

g mtry = 20

h mtry = 20

i mtry = 60

j mtry = 150

- 1. ****Excretion****

**CL and T_1/2_:** For these two properties, the percentage of compounds predicted within different fold error (Fold) was applied to assess model performance. They are defined as follows: fold= 1+|Y_pred_-Y_true_|/Y_true_. A prediction method with an average-fold error <2 was considered successful. The selected descriptors for CL and T_1/2_ were listed in Table 25 and Table 26 respectively and their statistic results were listed in Table 27. From this table, we can see that 76% of training compounds and 81.6% of test compounds are within 2-fold error for CL prediction. As to the T_1/2_ prediction, 76.2% of training compounds and 69.9% of test compounds are within 2-fold error. Corresponding fold-rate relationship can be seen in Figure 12 and Figure 13.

**Table 25.** Selected descriptors in CL modeling process

| **Descriptors (40)** |
| --- |
| nsulph, VSAEstate8, QNmin, IDET, ndb, slogPVSA2, MATSv5, S32, QCss, bcutm4, S9, bcutp8, Tnc, nsb, Geto, bcutp11, S7, MATSm2, GMTIV, nhet, MATSe1, CIC0, bcutp3, Gravto, EstateVSA9, MATSe3, MATSe5, UI, S53, J, bcute1, MRVSA9, PEOEVSA0, MATSv2, IDE, AWeight, IC0, S16, bcutp1, PEOEVSA12 |

**Table 26.** Selected descriptors in T_1/2_ modeling process

| **Descriptors (40)** |
| --- |
| MATSv5, Gravto, Chiv3c, PEOEVSA7, knotp, bcutp3, bcutm9, EstateVSA3, MATSp1, bcutp11, VSAEstate7, IC0, UI, Geto, QOmin, CIC0, dchi3, MATSp4, bcutm4, Hatov, MATSe4, CIC6, Chiv4, EstateVSA9, MATSv2, nring, bcute1, VSAEstate8, MRVSA9, PEOEVSA6, SIC1, bcutp8, MATSp6, QCss, J, IDE, CIC2, Hy, MRVSA6, naro, SPP, EstateVSA7, bcutv10, S12, LogP2, bcutp2, CIC3, S17, LogP, bcutp1 |

**Table 27**. The statistic result of CL and T_1/2_ models

| **Property** | **Method** | **Features** | **mtry** | **2-fold rate (CV/Test)** | **3-fold rate (CV/Test)** |
| --- | --- | --- | --- | --- | --- |
| CL | RF | 2D | 10 | 0.760/0.816 | 0.877/0.897 |
| T_1/2_ | RF | 2D | 12 | 0.762/0.699 | 0.897/0.824 |


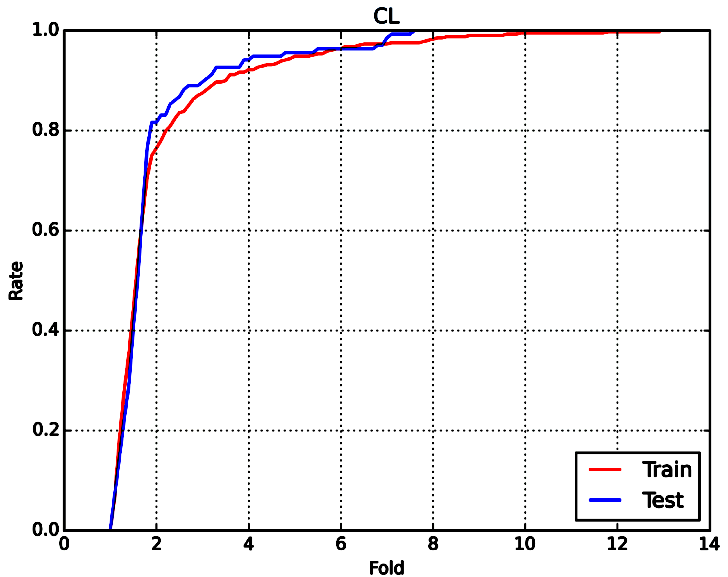


**Figure 12.** The fold-rate relationship of CL prediction


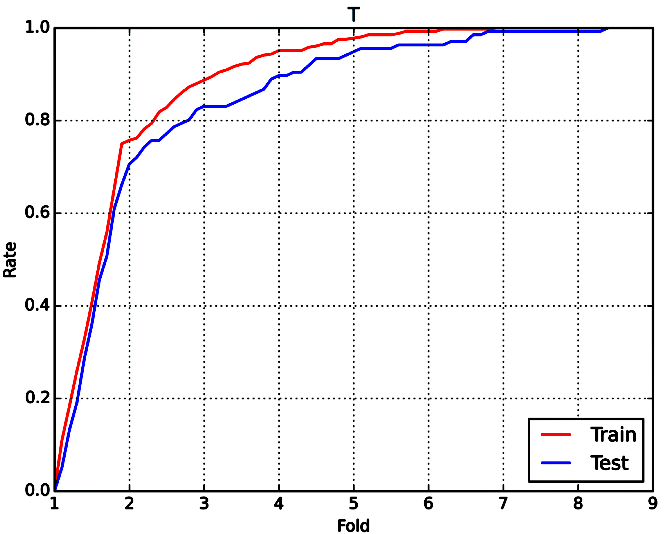


**Figure 13.** The fold-rate relationship of T_1/2_ prediction

- 1. ****Toxicity****

**hERG:** For this property, 20 classification models were developed by RF, SVM, and physicochemical 2D descriptors, MACCS, ECFP4, FP4. The statistic results for these classification models can be seen in Table 28. From the table 28, we can see that the classification model based on RF and 2D was the best one with ACC=0.844 for the training set and ACC=0.848 for the test set. Overall, our classification model has a comparable predictive ability compared with the latest study by Hou (ACC=84.7 for training set, ACC=82.1 for test set).[[65](#_ENREF_65)] The ROC curve of this classification model can be seen in Figure 14.

**Table 28**. The statistic results of different classification models

| Method | fingerprint | Five folds cross validation | | | | External validation dataset | | | |
| --- | --- | --- | --- | --- | --- | --- | --- | --- | --- |
|  |  | Sensitivity | Specificity | Accuracy | AUC | Sensitivity | Specificity | Accuracy | AUC |
| SVM | 2D | 0.912 | 0.617 | 0.821 | 0.861 | 0.849 | 0.702 | 0.802 | 0.861 |
|  | MACCS | 0.948 | 0.340 | 0.766 | 0.785 | 0.953 | 0.421 | 0.768 | 0.843 |
|  | FP4 | 0.852 | 0.497 | 0.745 | 0.740 | 0.916 | 0.596 | 0.805 | 0.829 |
|  | ECFP4 | 0.936 | 0.367 | 0.766 | 0.779 | 0.963 | 0.456 | 0.787 | 0.834 |
| RF | **2D** | **0.908** | **0.700** | **0.844** | **0.879** | **0.888** | **0.762** | **0.848** | **0.873** |
|  | MACCS | 0.913 | 0.510 | 0.792 | 0.860 | 0.944 | 0.632 | 0.835 | 0.880 |
|  | FP4 | 0.910 | 0.497 | 0.786 | 0.831 | 0.935 | 0.561 | 0.805 | 0.848 |
|  | ECFP4 | 0.913 | 0.422 | 0.766 | 0.806 | 0.944 | 0.526 | 0.799 | 0.857 |


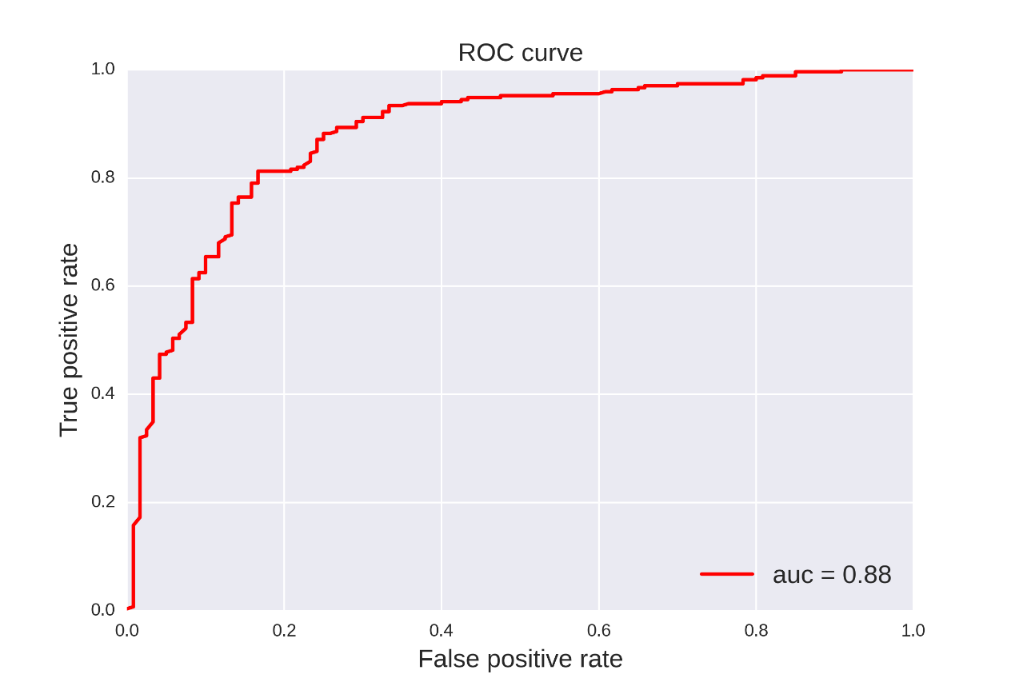


**Figure 14.** The ROC curve for the cross validation in training set.

**H-HT:** For this property, four classification models were developed by SVM, RF and physicochemical descriptors, FP4, MACCS, ECFP4. The statistic results for these classification models can be seen in Table 29. From the table 29, we can see that the classification model based on RF and physicochemical 2D descriptor was the best one with ACC=0.689 for the training set and ACC=0.681 for the test set. Compared with the similar study in 2015 (AUC=0.73, ACC=0.75), our new classification model has a comparable statistic result and may help to detect human hepatotoxicity in drug discovery process.[[66](#_ENREF_66)] The ROC curve of this classification model can be seen in Figure 15.

**Table 29**. The statistic results of different classification models

| Method | fingerprint | Five folds cross validation | | | | External validation dataset | | | |
| --- | --- | --- | --- | --- | --- | --- | --- | --- | --- |
|  |  | Sensitivity | Specificity | Accuracy | AUC | Sensitivity | Specificity | Accuracy | AUC |
| SVM | 2D | 0.742 | 0.493 | 0.658 | 0.660 | 0.746 | 0.466 | 0.648 | 0.636 |
|  | MACCS | 0.858 | 0.431 | 0.715 | 0.701 | 0.873 | 0.386 | 0.703 | 0.712 |
|  | FP4 | 0.862 | 0.450 | 0.724 | 0.745 | 0.856 | 0.471 | 0.722 | 0.729 |
|  | ECFP4 | 0.903 | 0.302 | 0.701 | 0.703 | 0.907 | 0.328 | 0.705 | 0.718 |
| RF | **2D** | **0.776** | **0.520** | **0.689** | **0.710** | **0.785** | **0.487** | **0.681** | **0.683** |
|  | MACCS | 0.875 | 0.395 | 0.714 | 0.722 | 0.870 | 0.397 | 0.705 | 0.723 |
|  | FP4 | 0.862 | 0.463 | 0.728 | 0.743 | 0.853 | 0.487 | 0.726 | 0.753 |
|  | ECFP4 | 0.908 | 0.311 | 0.708 | 0.720 | 0.918 | 0.339 | 0.716 | 0.726 |


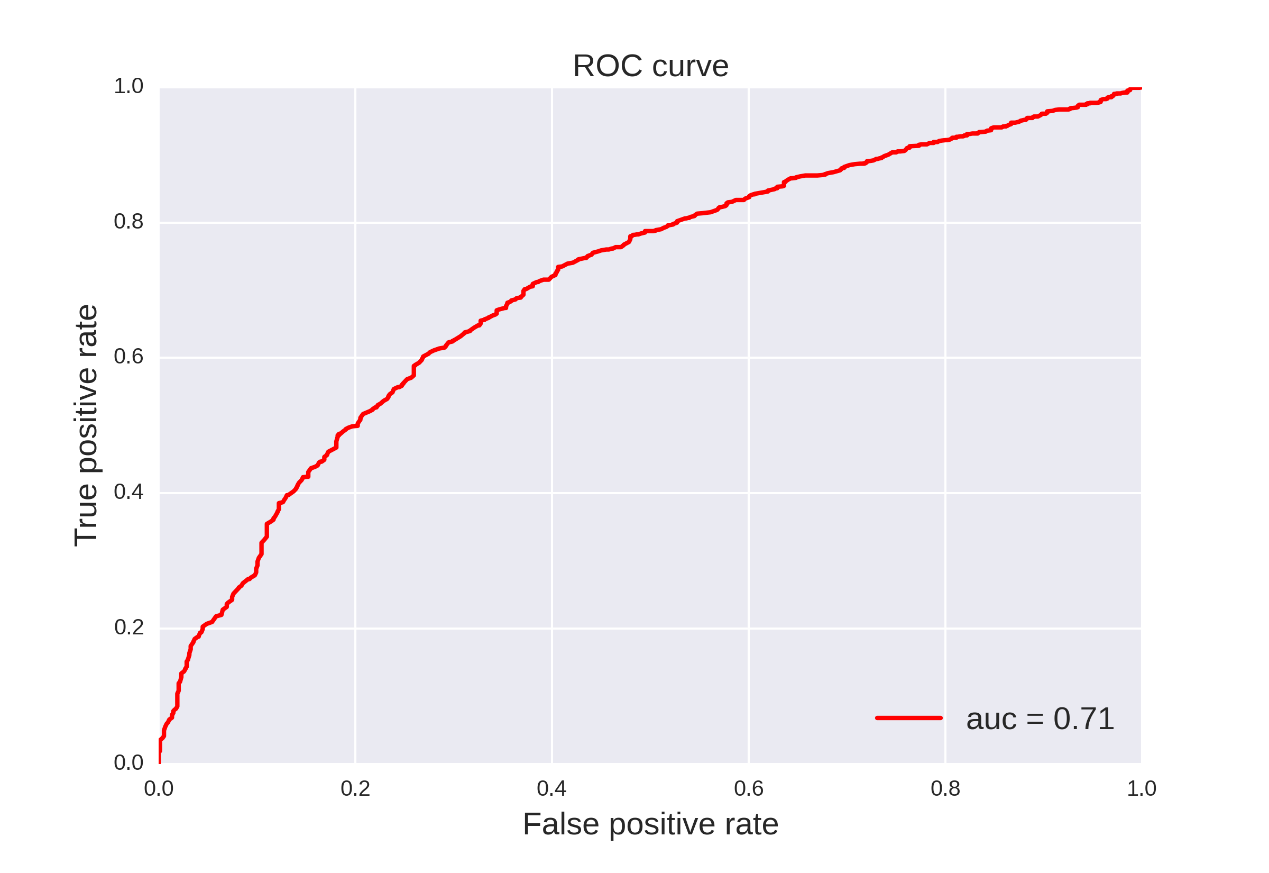


**Figure 15.** The ROC curve for the cross validation in training set.

**Ames:** For this property, four classification models were developed by RF and Estate, MACCS, FP4 and ECFP4. The statistic results for these classification models can be seen in Table 30. From the table 30, we can see that the classification model based on RF and MACCS was the best one with ACC=0.820 for the training set and ACC=0.834 for the test set. In 2012, Congying Xu developed a series of classification models and the best one has a ACC value of 0.841 and a AUC value of 0.901. Compared with it, our prediction model has a comparable result and will be useful in practical application.[[67](#_ENREF_67)] The ROC curve of this classification model can be seen in Figure 16.

**Table 30**. The statistic results of different classification models

| Method | fingerprint | Five folds cross validation | | | | External validation dataset | | | |
| --- | --- | --- | --- | --- | --- | --- | --- | --- | --- |
|  |  | Sensitivity | Specificity | Accuracy | AUC | Sensitivity | Specificity | Accuracy | AUC |
| RF | Estate | 0.818 | 0.784 | 0.803 | 0.872 | 0.834 | 0.794 | 0.817 | 0.882 |
|  | **MACCS** | **0.834** | **0.800** | **0.820** | **0.890** | **0.816** | **0.848** | **0.834** | **0.897** |
|  | FP4 | 0.849 | 0.683 | 0.775 | 0.840 | 0.847 | 0.685 | 0.775 | 0.846 |
|  | ECFP4 | 0.828 | 0.794 | 0.813 | 0.890 | 0.840 | 0.787 | 0.817 | 0.899 |


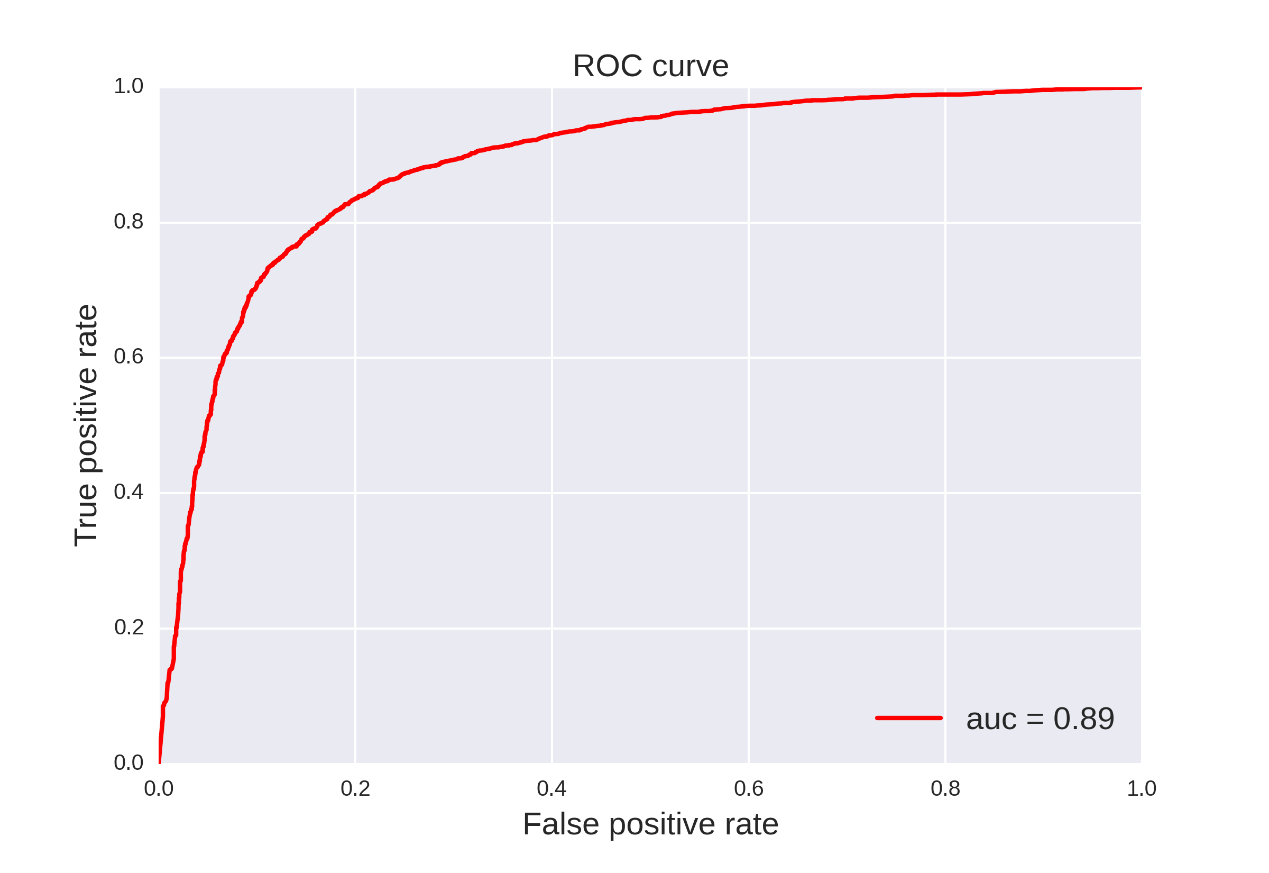


**Figure 16.** The ROC curve for the cross validation in training set.

# Summary

In this study, we built a series of QSAR models for ADME/T related properties based on different descriptors and different methods. For each property, we chose a best model according to their statistic results and these best models were listed in Table 31 and Table 32.

**Table 31.** The best regression models for some ADME/T related properties

| **Property** | **Method** | **mtry** | **R^2^** | **Q^2^** | **R^2^_T_** | **RMSE_F_** | **RMSE_CV_** | **RMSE_T_** |  |
| --- | --- | --- | --- | --- | --- | --- | --- | --- | --- |
| LogS | RF | 10 | 0.980 | 0.860 | 0.979 | 0.095 | 0.698 | 0.712 |  |
| LogD_7.4_ | RF | 14 | 0.983 | 0.877 | 0.874 | 0.228 | 0.614 | 0.605 |  |
| Caco-2 | RF | 14 | 0.973 | 0.845 | 0.824 | 0.121 | 0.289 | 0.290 |  |
| PPB | RF | - | 0.954 | 0.691 | 0.682 | 7.124 | 18.443 | 18.044 |  |

| **Property** | **Method** | **Features** | **mtry** | **2-fold rate (CV/Test)** | **3-fold rate (CV/Test)** |
| --- | --- | --- | --- | --- | --- |
| VD | RF | 2D | 10 | 0.819/0.801 | 0.912/0.904 |
| CL | RF | 2D | 10 | 0.760/0.816 | 0.877/0.897 |
| T_1/2_ | RF | 2D | 12 | 0.762/0.699 | 0.897/0.824 |
| LD50 | RF | 2D | 5 | 0.986/0.987 | 0.998/0.997 |

**Table 32.** The best classification models for some ADME/T related properties

| **Property** | **Method** | **Features** | **Five-fold cross validation** | | | | | | **External validation dataset** | | | | | | | |
| --- | --- | --- | --- | --- | --- | --- | --- | --- | --- | --- | --- | --- | --- | --- | --- | --- |
|  |  |  | Sensitivity | Specificity | Accuracy | | AUC | | Sensitivity | | Specificity | | Accuracy | | AUC | |
| HIA | RF | MACCS | 0.820 | 0.743 | 0.782 | | 0.846 | | 0.801 | | 0.743 | | 0.773 | | 0.831 | |
| F (20%) | RF | MACCS | 0.731 | 0.647 | 0.689 | | 0.759 | | 0.680 | | 0.663 | | 0.671 | | 0.746 | |
| F (30%) | RF | ECFP6 | 0.743 | 0.605 | 0.669 | | 0.715 | | 0.751 | | 0.601 | | 0.667 | | 0.718 | |
| BBB | SVM | ECFP2 | 0.962 | 0.813 | | 0.926 | | 0.948 | | 0.993 | | 0.854 | | 0.962 | | 0.975 |
| Pgp-inhibitor | SVM | ECFP4 | 0.887 | 0.789 | | 0.848 | | 0.908 | | 0.863 | | 0.802 | | 0.838 | | 0.913 |
| Pgp-substrate | SVM | ECFP4 | 0.839 | 0.807 | | 0.824 | | 0.899 | | 0.826 | | 0.854 | | 0.840 | | 0.905 |
| CYP1A2-Inhibitor | SVM | ECFP4 | 0.833 | 0.864 | | 0.849 | | 0.928 | | 0.853 | | 0.880 | | 0.867 | | 0.939 |
| CYP1A2-Substrate | RF | ECFP4 | 0.768 | 0.636 | | 0.702 | | 0.801 | | 0.768 | | 0.637 | | 0.702 | | 0.802 |
| CYP3A4-Inhibitor | SVM | ECFP4 | 0.759 | 0.858 | | 0.817 | | 0.901 | | 0.788 | | 0.860 | | 0.829 | | 0.909 |
| CYP3A4-Substrate | RF | ECFP4 | 0.798 | 0.716 | | 0.757 | | 0.835 | | 0.819 | | 0.679 | | 0.749 | | 0.835 |
| CYP2C19-Inhibitor | SVM | ECFP2 | 0.826 | 0.819 | | 0.822 | | 0.893 | | 0.812 | | 0.825 | | 0.819 | | 0.899 |
| CYP2C19-Substrate | RF | ECFP2 | 0.735 | 0.744 | | 0.740 | | 0.816 | | 0.871 | | 0.667 | | 0.769 | | 0.853 |
| CYP2C9-Inhibitor | SVM | ECFP4 | 0.719 | 0.898 | | 0.837 | | 0.900 | | 0.730 | | 0.882 | | 0.830 | | 0.894 |
| CYP2C9-Substrate | RF | ECFP4 | 0.746 | 0.709 | | 0.728 | | 0.819 | | 0.746 | | 0.709 | | 0.734 | | 0.824 |
| CYP2D6-Inhibitor | RF | ECFP4 | 0.770 | 0.811 | | 0.793 | | 0.868 | | 0.771 | | 0.812 | | 0.795 | | 0.882 |
| CYP2D6-Substrate | RF | ECFP4 | 0.765 | 0.73 | | 0.748 | | 0.823 | | 0.792 | | 0.73 | | 0.76 | | 0.833 |
| hERG | RF | 2D | 0.908 | 0.700 | | 0.844 | | 0.879 | | 0.888 | | 0.762 | | 0.848 | | 0.873 |
| H-HT | RF | 2D | 0.780 | 0.520 | | 0.689 | | 0.710 | | 0.785 | | 0.487 | | 0.681 | | 0.683 |
| Ames | RF | MACCS | 0.800 | 0.841 | | 0.820 | | 0.890 | | 0.848 | | 0.816 | | 0.834 | | 0.897 |
| SkinSen | RF | MACCS | 0.685 | 0.727 | | 0.706 | | 0.760 | | 0.715 | | 0.727 | | 0.731 | | 0.774 |
| DILI | RF | MACCS | 0.866 | 0.813 | | 0.840 | | 0.904 | | 0.830 | | 0.857 | | 0.843 | | 0.910 |
| FDAMDD | RF | ECFP4 | 0.848 | 0.812 | | 0.832 | | 0.904 | | 0.853 | | 0.782 | | 0.821 | | 0.892 |

# Reference

1. Prentis, R.A., Y. Lis, and S.R. Walker, *Pharmaceutical innovation by the seven UK-owned pharmaceutical companies (1964-1985).* British Journal of Clinical Pharmacology, 1988. **25**(3): p. 387-96.

2. Kennedy, T., *Managing the drug discovery/development interface.* Drug Discovery Today, 1997. **2**(10): p. 436-444.

3. Han, V.D.W. and E. Gifford, *ADMET in silico modelling: towards prediction paradise?* Nature Reviews Drug Discovery, 2003. **2**(3): p. 192-204.

4. Wang, Y., et al., *In silico ADME/T modelling for rational drug design.* Quarterly Reviews of Biophysics, 2015. **-1**(4): p. 1-28.

5. Huuskonen†, J., *Estimation of Aqueous Solubility for a Diverse Set of Organic Compounds Based on Molecular Topology.* ChemInform, 2000. **40**(35): p. 773-7.

6. Delaney, J.S., *ESOL: estimating aqueous solubility directly from molecular structure.* Journal of Chemical Information & Computer Sciences, 2004. **44**(3): p. 1000-5.

7. Waring, M.J., *Lipophilicity in drug discovery.* Expert Opinion on Drug Discovery, 2010. **5**(3): p. 235-48.

8. Arnott, J.A. and S.L. Planey, *The influence of lipophilicity in drug discovery and design.* Expert Opinion on Drug Discovery, 2012. **7**(10): p. 863-75.

9. Testa, B., et al., *The influence of lipophilicity on the pharmacokinetic behavior of drugs: Concepts and examples.* Perspectives in Drug Discovery and Design, 2000. **19**(1): p. 179-211.

10. Wang, J.B., et al., *In silico evaluation of logD 7.4 and comparison with other prediction methods.* Journal of Chemometrics, 2015. **29**(7): p. 389–398.

11. Lin, J., et al., *The role of absorption, distribution, metabolism, excretion and toxicity in drug discovery.* Current Topics in Medicinal Chemistry, 2003. **3**(10): p. 1125-54.

12. Artursson, P., K. Palm, and K. Luthman, *Caco-2 monolayers in experimental and theoretical predictions of drug transport.* Advanced Drug Delivery Reviews, 1996. **22**(1–2): p. 67-84.

13. Sun, H. and K.S. Pang, *Permeability, transport, and metabolism of solutes in caco-2 cell monolayers: A theoretical study.* Drug Metabolism & Disposition the Biological Fate of Chemicals, 2008. **36**(1): p. 102-23.

14. Wang, N.N., et al., *ADME Properties Evaluation in Drug Discovery: Prediction of Caco-2 Cell Permeability Using a Combination of NSGA-II and Boosting.* Journal of Chemical Information & Modeling, 2016. **56**(4).

15. F, B., et al., *A novel approach for predicting P-glycoprotein (ABCB1) inhibition using molecular interaction fields.* Journal of Medicinal Chemistry, 2011. **54**(6): p. 1740-51.

16. Shugarts, S. and L.Z. Benet, *The Role of Transporters in the Pharmacokinetics of Orally Administered Drugs.* Pharmaceutical Research, 2009. **26**(9): p. 2039-2054.

17. Chen, L., et al., *Computational models for predicting substrates or inhibitors of P-glycoprotein.* Drug Discovery Today, 2011. **17**(7-8): p. 343-51.

18. Chen, L., et al., *ADME evaluation in drug discovery. 10. Predictions of P-glycoprotein inhibitors using recursive partitioning and naive Bayesian classification techniques.* Molecular Pharmaceutics, 2011. **8**(3): p. 889-900.

19. Wang, Z., et al., *P-glycoprotein substrate models using support vector machines based on a comprehensive data set.* Journal of Chemical Information & Modeling, 2011. **51**(6): p. 1447-56.

20. Li, D., et al., *ADMET evaluation in drug discovery. 13. Development of in silico prediction models for P-glycoprotein substrates.* Molecular Pharmaceutics, 2014. **11**(3): p. 716.

21. Hou, T., et al., *ADME evaluation in drug discovery. 7. Prediction of oral absorption by correlation and classification.* ChemInform, 2007. **47**(15): p. 208-18.

22. Hou, T., J. Wang, and Y. Li, *ChemInform Abstract: ADME Evaluation in Drug Discovery. Part 8. The Prediction of Human Intestinal Absorption by a Support Vector Machine.* ChemInform, 2008. **47**(8): p. 2408-2415.

23. Wang, N.-N., et al., *Predicting human intestinal absorption with modified random forest approach: a comprehensive evaluation of molecular representation, unbalanced data, and applicability domain issues.* RSC Advances, 2017. **7**(31): p. 19007-19018.

24. Tian, S., et al., *ADME Evaluation in Drug Discovery. 9. Prediction of Oral Bioavailability in Humans Based on Molecular Properties and Structural Fingerprints.* Molecular Pharmaceutics, 2011. **8**(3): p. 841-51.

25. Ma, C.Y., et al., *Prediction models of human plasma protein binding rate and oral bioavailability derived by using GA-CG-SVM method.* Journal of Pharmaceutical & Biomedical Analysis, 2008. **47**(4-5): p. 677-82.

26. Zhu, X.W., et al., *The use of pseudo-equilibrium constant affords improved QSAR models of human plasma protein binding.* Pharmaceutical Research, 2013. **30**(7): p. 1790-8.

27. Hall, L.M., L.H. Hall, and L.B. Kier, *QSAR modeling of beta-lactam binding to human serum proteins.* Journal of Computer-Aided Molecular Design, 2003. **17**(2): p. 103-18.

28. Zhivkova, Z. and I. Doytchinova, *Quantitative structure—plasma protein binding relationships of acidic drugs.* Journal of Pharmaceutical Sciences, 2012. **101**(12): p. 4627-4641.

29. Ghafourian, T. and Z. Amin, *QSAR Models for the Prediction of Plasma Protein Binding.* Bioimpacts, 2013. **3**(1): p. 21-7.

30. Norinder, U. and M. Haeberlein, *Computational approaches to the prediction of the blood-brain distribution.* Advanced Drug Delivery Reviews, 2002. **54**(3): p. 291-313.

31. Dayan, A.D. and A.D. Dayan, *Book Reviews : Goodman & Gilman's The Pharmacological Basis of Therapeutics - Ninth EditionJoel G Hardman, Lee E Limbird (Editors-in-Chief), Perry B Molinoff and Raymond W Ruddon, Editors and Alfred Goodman Gilman, Consulting Editor, McGraw-Hill Health Pro.* Human & Experimental Toxicology, 1996. **15**(7): p. 605-605.

32. Li, H., et al., *Effect of selection of molecular descriptors on the prediction of blood-brain barrier penetrating and nonpenetrating agents by statistical learning methods.* Journal of Chemical Information & Modeling, 2005. **45**(5): p. 1376-84.

33. Shen, J., et al., *Estimation of ADME properties with substructure pattern recognition.* Journal of Chemical Information & Modeling, 2010. **50**(6): p. 1034-41.

34. Nicholson, J.K., et al., *Host-Gut Microbiota Metabolic Interactions.* Science, 2012. **336**(6086): p. 1262-7.

35. Kirchmair, J., et al., *Predicting drug metabolism: experiment and/or computation?* Nat Rev Drug Discov, 2015. **14**(6): p. 387-404.

36. Veith, H., et al., *Comprehensive characterization of cytochrome P450 isozyme selectivity across chemical libraries.* Nature Biotechnology, 2009. **27**(11): p. 1050-5.

37. Rostkowski, M., O. Spjuth, and P. Rydberg, *WhichCyp: prediction of cytochromes P450 inhibition.* Bioinformatics, 2013. **29**(16): p. 2051-2.

38. Carbon-Mangels, M. and M.C. Hutter, *Selecting Relevant Descriptors for Classification by Bayesian Estimates: A Comparison with Decision Trees and Support Vector Machines Approaches for Disparate Data Sets.* Molecular Informatics, 2011. **30**(10): p. 885–895.

39. Zaretzki, J., M. Matlock, and S.J. Swamidass, *XenoSite: accurately predicting CYP-mediated sites of metabolism with neural networks.* Journal of Chemical Information & Modeling, 2013. **53**(12): p. 3373-83.

40. Obach, R.S., F. Lombardo, and N.J. Waters, *Trend analysis of a database of intravenous pharmacokinetic parameters in humans for 670 drug compounds.* Drug Metabolism & Disposition the Biological Fate of Chemicals, 2008. **36**(7): p. 1385-405.

41. Smith, P.L., T. Baukrowitz, and G. Yellen, *The inward rectification mechanism of the HERG cardiac potassium channel.* Nature, 1996. **379**(379): p. 833-6.

42. Vandenberg, J.I., et al., *hERG K(+) channels: structure, function, and clinical significance.* Physiological Reviews, 2012. **92**(3): p. 1393-1478.

43. Wang, S., et al., *ADMET Evaluation in Drug Discovery. 16. Predicting hERG Blockers by Combining Multiple Pharmacophores and Machine Learning Approaches.* Molecular Pharmaceutics, 2016. **13**(8).

44. Man, F., et al., *Evaluation of the Characteristics of Safety Withdrawal of Prescription Drugs from Worldwide Pharmaceutical Markets1960 to 1999.* Therapeutic Innovation & Regulatory Science, 2001. **35**(1): p. 293-317.

45. Ballet, F., *Hepatotoxicity in drug development: detection, significance and solutions.* Journal of Hepatology, 1997. **26 Suppl 2**(26 Suppl 2): p. 26-36.

46. Mulliner, D., et al., *Computational Models for Human and Animal Hepatotoxicity with a Global Application Scope.* Chemical Research in Toxicology, 2016.

47. Ames, B.N., J. Mccann, and E. Yamasaki, *Methods for detecting carcinogens and mutagens with the salmonella/mammalian-microsome mutagenicity test ☆.* Mutation Research/fundamental & Molecular Mechanisms of Mutagenesis, 1975. **31**(6): p. 347-64.

48. Sushko, I., et al., *Applicability domain for in silico models to achieve accuracy of experimental measurements.* Journal of Chemometrics, 2010. **24**(3-4): p. 202–208.

49. Benigni, R. and A. Giuliani, *Computer-assisted analysis of interlaboratory Ames test variability.* Journal of Toxicology & Environmental Health, 1988. **25**(1): p. 135-48.

50. Xu, C., et al., *In silico prediction of chemical Ames mutagenicity.* Journal of Chemical Information & Modeling, 2012. **52**(11): p. 2840-7.

51. Alves, V.M., et al., *Predicting chemically-induced skin reactions. Part I: QSAR models of skin sensitization and their application to identify potentially hazardous compounds.* Toxicology & Applied Pharmacology, 2015. **284**(2): p. 262-272.

52. Lei, T., et al., *ADMET evaluation in drug discovery: 15. Accurate prediction of rat oral acute toxicity using relevance vector machine and consensus modeling.* Journal of Cheminformatics, 2016. **8**: p. 6.

53. Xu, Y., et al., *Deep Learning for Drug-Induced Liver Injury.* Journal of Chemical Information & Modeling, 2015. **55**(10): p. 2085.

54. Cao, D.S., et al., *In silico toxicity prediction of chemicals from EPA toxicity database by kernel fusion-based support vector machines.* Chemometrics & Intelligent Laboratory Systems, 2015. **146**: p. 494-502.

55. Salahinejad, M., T.C. Le, and D.A. Winkler, *Aqueous solubility prediction: do crystal lattice interactions help?* Molecular Pharmaceutics, 2013. **10**(7): p. 2757.

56. Wang, J.B., et al., *In silico evaluation of logD7.4 and comparison with other prediction methods.* Journal of Chemometrics, 2015. **29**(7): p. 389-398.

57. Wang, N.N., et al., *ADME Properties Evaluation in Drug Discovery: Prediction of Caco-2 Cell Permeability Using a Combination of NSGA-II and Boosting.* Journal of Chemical Information & Modeling, 2016. **56**(4): p. 763.

58. Chen, L., et al., *ADME Evaluation in Drug Discovery. 10. Predictions of P-Glycoprotein Inhibitors Using Recursive Partitioning and Naive Bayesian Classification Techniques.* Mol Pharm, 2011. **8**(3): p. 889-900.

59. Wang, N.N., et al., *Predicting human intestinal absorption with modified random forest approach: a comprehensive evaluation of molecular representation, unbalanced data, and applicability domain issues.* Rsc Advances, 2017. **7**(31): p. 19007-19018.

60. Wang, J. and T. Hou, *Advances in computationally modeling human oral bioavailability.* Advanced Drug Delivery Reviews, 2015. **86**(Supplement C): p. 11-16.

61. Wang, N.N., et al., *ADME properties evaluation in drug discovery: Prediction of plasma protein binding using NSGA-II combining PLS and consensus modeling.* Chemometrics & Intelligent Laboratory Systems, 2017.

62. Berellini, G., et al., *In Silico Prediction of Volume of Distribution in Human Using Linear and Nonlinear Models on a 669 Compound Data Set.* Journal of Medicinal Chemistry, 2009. **52**(14): p. 4488.

63. Li, H., et al., *Effect of selection of molecular descriptors on the prediction of blood-brain barrier penetrating and nonpenetrating agents by statistical learning methods.* Journal of Chemical Information & Modeling, 2005. **45**(5): p. 1376.

64. Daina, A., O. Michielin, and V. Zoete, *SwissADME: a free web tool to evaluate pharmacokinetics, drug-likeness and medicinal chemistry friendliness of small molecules.* Scientific Reports, 2017. **7**: p. 42717.

65. Wang, S., et al., *ADMET Evaluation in Drug Discovery. 16. Predicting hERG Blockers by Combining Multiple Pharmacophores and Machine Learning Approaches.* Molecular Pharmaceutics, 2016. **13**(8): p. 2855.

66. Mulliner, D., et al., *Computational Models for Human and Animal Hepatotoxicity with a Global Application Scope.* Chemical Research in Toxicology, 2016. **29**(5): p. 757.

67. Xu, C., et al., *In silico Prediction of Chemical Ames Mutagenicity.* Journal of Chemical Information & Modeling, 2012. **52**(11): p. 2840.
